# Rapid evaporative ionisation mass spectrometry of electrosurgical vapours for the identification of breast pathology: towards an intelligent knife for breast cancer surgery

**DOI:** 10.1186/s13058-017-0845-2

**Published:** 2017-05-23

**Authors:** Edward R. St John, Julia Balog, James S. McKenzie, Merja Rossi, April Covington, Laura Muirhead, Zsolt Bodai, Francesca Rosini, Abigail V. M. Speller, Sami Shousha, Rathi Ramakrishnan, Ara Darzi, Zoltan Takats, Daniel R. Leff

**Affiliations:** 1 Department of BioSurgery and Surgical Technology, Imperial College London, London, UK; 2 Division of Computational and Systems Medicine, Imperial College, London, UK; 3 Waters Research Centre, Budapest, Hungary; 4 Department of Pathology, Imperial College NHS Trust, London, UK; 5 Sir Alexander Fleming Building, South Kensington Campus, Imperial College, London, SW7 2AZ UK; 6 Department of BioSurgery and Surgical Technology, Clinical Senior Lecturer and Consultant Breast Surgeon, St Mary’s Hospital, 10th Floor, QEQM Wing, London, W2 1NY UK

**Keywords:** Breast, Cancer, Margins, Intraoperative margin assessment, Surgery, Mass spectrometry, Rapid evaporative ionisation mass spectrometry, REIMS, Intelligent knife, iKnife

## Abstract

**Background:**

Re-operation for positive resection margins following breast-conserving surgery occurs frequently (average = 20–25%), is cost-inefficient, and leads to physical and psychological morbidity. Current margin assessment techniques are slow and labour intensive. Rapid evaporative ionisation mass spectrometry (REIMS) rapidly identifies dissected tissues by determination of tissue structural lipid profiles through on-line chemical analysis of electrosurgical aerosol toward real-time margin assessment.

**Methods:**

Electrosurgical aerosol produced from ex-vivo and in-vivo breast samples was aspirated into a mass spectrometer (MS) using a monopolar hand-piece. Tissue identification results obtained by multivariate statistical analysis of MS data were validated by histopathology. Ex-vivo classification models were constructed from a mass spectral database of normal and tumour breast samples. Univariate and tandem MS analysis of significant peaks was conducted to identify biochemical differences between normal and cancerous tissues. An ex-vivo classification model was used in combination with bespoke recognition software, as an intelligent knife (iKnife), to predict the diagnosis for an ex-vivo validation set. Intraoperative REIMS data were acquired during breast surgery and time-synchronized to operative videos.

**Results:**

A classification model using histologically validated spectral data acquired from 932 sampling points in normal tissue and 226 in tumour tissue provided 93.4% sensitivity and 94.9% specificity. Tandem MS identified 63 phospholipids and 6 triglyceride species responsible for 24 spectral differences between tissue types. iKnife recognition accuracy with 260 newly acquired fresh and frozen breast tissue specimens (normal *n* = 161, tumour *n* = 99) provided sensitivity of 90.9% and specificity of 98.8%. The ex-vivo and intra-operative method produced visually comparable high intensity spectra. iKnife interpretation of intra-operative electrosurgical vapours, including data acquisition and analysis was possible within a mean of 1.80 seconds (SD ±0.40).

**Conclusions:**

The REIMS method has been optimised for real-time iKnife analysis of heterogeneous breast tissues based on subtle changes in lipid metabolism, and the results suggest spectral analysis is both accurate and rapid. Proof-of-concept data demonstrate the iKnife method is capable of online intraoperative data collection and analysis. Further validation studies are required to determine the accuracy of intra-operative REIMS for oncological margin assessment.

**Electronic supplementary material:**

The online version of this article (doi:10.1186/s13058-017-0845-2) contains supplementary material, which is available to authorized users.

## Background

Breast cancer is the commonest cancer in women, with the fifth highest mortality rates worldwide [1]. Breast conserving surgery (BCS) is the most commonly performed surgical technique for the treatment of women with early stage breast cancer in both the United States of America (USA) and the United Kingdom (UK) [2, 3]. On both sides of the Atlantic, over a fifth of patients undergoing BCS require re-operation for inadequate margins [3–7]. Meta-analytical data suggest that a positive resection margin following BCS more than doubles the chance of ipsilateral breast tumour regional recurrence (IBTR) [8]. The risk of relapse is not eliminated by the use of radiotherapy, systemic chemotherapy or endocrine therapy [9].

Re-operation is associated with physical and psychological morbidity and has economic implications. Re-operative intervention is associated with greater patient anxiety, impaired cosmesis [10] and a higher incidence of post-operative wound complications [11], and may delay receipt of adjuvant therapies [12]. Moreover, re-operation increases healthcare costs. For example, an economic model of re-excision of breast margins in the USA predicted that in comparison to positive margins, re-excision of close margins (<2 mm) accounts for an additional US$18.8 million per year whilst eliminating re-excision of margins ultimately found to be negative would save a further US$16.4 million per year [13].

Intraoperative margin assessment (IMA) techniques aim to provide the surgeon with actionable information about margin status in the midst of the index procedure to reduce the need for re-operation. Pathological techniques, frozen section and cytology (i.e. imprint, touch and scrape) are demonstrably accurate [14] and may reduce re-operation rates for positive margins [15, 16]. For example, frozen section has been demonstrated to significantly reduce reoperation rates from 13.2% to 3.6% [17] and its uptake may be cost-effective [18]. However, the limitations of pathological techniques, including slow turnaround times, manpower requirements, and the potential for false positive interpretation, have limited international adoption. Specimen radiology (SR) and intraoperative ultrasound (IOUS) can be used to assess margin status and can be performed within the operating theatre providing direct feedback to the surgeon without the need for specially trained personnel [19, 20]. However, compared to pathological techniques they have inferior accuracy [14], and hence do not parallel the observed reductions in re-operation rates [21].

Due to the limitations of contemporary IMA techniques a plethora of innovative devices are under development aiming to provide a tool that limits workflow disruption, provides rapid results, optimizes margincontrol and reduces re-operation rates. A variety of imaging and probe-based devices are emerging that are designed to detect differences in tissue properties between cancerous and normal breast tissue [22]. Bioimpedance is the measure of the response of tissue to an externally applied electrical current; the MarginProbe™ is quick (~5–7 minutes) and a 50% reduction in re-operation rates is achievable despite modest sensitivity and specificity (~70%) [23, 24]. Similarly, the ClearEdge™ system measures tissue-specific electrical properties with promising preliminary data (sensitivity ~85%, specificity ~80%) [25]. Optical spectroscopy techniques such as diffuse reflectance [26–28], Raman spectroscopy [29], optical coherence tomography (OCT) [30], spatial frequency domain imaging [31], fluorescence techniques [32] and confocal microscopy [33] all measure tissue response to light at various wavelengths, and whilst preliminary results are promising [34], the diagnostic accuracy of the techniques is inferior to pathological margin assessment and technological developments are required to increase image processing time and improve the ease of use.

Mass spectrometry (MS) is an innovative addition to the field of margin detection technologies. Molecules are analysed by measuring the mass-to-charge ratio (*m/z*) of molecular ions and their charged fragments. MS is well-established as a tool for quantifying small molecules and is also valuable for identifying metabolites and biomarkers [35, 36]. Over the last decade, advances in MS instrumentation have resulted in the ability to detect proteins and metabolites directly from tissues via imaging applications. A variety of MS platforms including matrix-assisted laser desorption/ionisation (MALDI) [37] and desorption electrospray ionisation (DESI) [38] show promise in differentiating tissue types with potential applications in rapid tissue diagnostics [39].

Rapid evaporative ionisation mass spectrometry (REIMS) [40–42] is an ambient ionisation technique that utilizes the aerosol by-product of electrosurgical (Bovie) tools. The electrosurgical process of cutting (i.e. continuous radiofrequency (RF) wave) or coagulating (i.e. pulsed RF wave) tissue causes heat to dissipate inside the tissue resulting in cellular explosion and release of cellular content to the gas phase. Aspiration of the aerosol allows for rapid mass spectrometric chemical analysis and computational algorithms can “learn” (cf. ‘machine-learning’) to recognise the chemical differences between tissue types. The technology can identify tissue characteristics within a few seconds of electrosurgical activation [41].

There is clearly a need to develop a reliable, effective, and rapid IMA method for neoplastic tissue characterization with accuracy competitive with standard histological assessment that can guide resection in vivo and that improves quality in breast surgical oncology. The REIMS system or intelligent knife (iKnife), capable of providing intuitive feedback on real-time tissue characterisation at the point of dissection, offers a potential solution. Here, we test the hypothesis that malignant breast tissues exhibit different metabolic profiles compared to normal breast tissues, and that these changes can be exploited using REIMS. Finally, we demonstrate proof of concept that the iKnife method is capable of intra-operative analysis of electrosurgical vapours.

## Methods

A single-centre, prospective observational study was performed at Imperial College Healthcare NHS Trust (London, UK). Ethical approval was gained from South East London Research Ethics Committee Reference 11/LO/0686, the East of England - Cambridge East Research Ethics Committee Reference 14/EE/0024 and the project was registered under the Imperial College Tissue Bank. Patients (>18 years of age) undergoing breast surgery for benign and malignant disease were recruited. Data were only obtained on patients who had consented to utilization of tissue for research.

Demographic and clinicopathological details included age, operation type, neoadjuvant treatment, post-operative histopathological data including grade (1–3), tumour histological subtype (invasive ductal carcinoma (IDC), invasive lobular carcinoma (ILC), invasive mucinous carcinoma (IMC) or ductal carcinoma in situ (DCIS)), oestrogen receptor (ER) and progesterone receptor (PR) status and human epidermal growth factor receptor 2 (HER2) status (Additional file 1: Table S1). Tumours had to be of a macroscopic size ≥2 cm to allow for adequate research tissue without compromising the clinical diagnosis. Where feasible, tissue was provided from the centre of the tumour from non-necrotic areas. Normal tissue was obtained from patients without malignancy or at a site distant from the tumour specimen. Mass-forming DCIS was suitable for inclusion; however, patients with non-mass-forming DCIS were excluded because the provision of tissue risked compromising the diagnosis.

### MS instrument and ex-vivo analysis workflow

Breast tissue samples were trimmed to size (3–10 mm^2^) and between 1 and 10 small cuts were made through the tissue using a modified monopolar blade electrosurgical pencil (Medres, Hungary) in either the pure cut setting (continuous RF wave) or fulgurate coag (pulsed RF wave) setting with a ForceTriad™ generator (Medtronic, Ireland). The power setting of the device varied between 10 and 30 W depending on the size and type of tissue. Aerosol produced as a result of electrosurgical activation was aspirated via the electrosurgical hand-piece [41] and transferred through a plastic tube to the mass spectrometer using a Venturi air jet pump. Surgical aerosol was co-aspirated with propan-2-ol (Sigma, MO, USA) (0.2 ml/minute) into the vacuum system of the Xevo G2-XS quadrupole time-of-flight mass spectrometer (product use is investigational) (Waters, UK). Aerosol particles and solvent droplets were de-clustered using a heated jet disruptor surface in the coarse vacuum regime of the instrument. Gaseous negative ions then entered the MS ion optics and were subjected to mass analysis. The remaining tissue was transferred to histology cassettes and sent to the pathology laboratory to be formalin fixed, paraffin embedded, sectioned and stained with haematoxylin and eosin (H&E). Subsequently, H&E-stained slides were examined by senior histopathologists to identify the tissue surrounding the sampling point and assign a tissue diagnosis (i.e. B1 = normal, B2 = benign, B3 = benign with uncertain malignant potential, B4 = suspicious, B5a = in-situ, or B5b = invasive tumour) according to the UK Royal College of Pathologists guidelines for non-operative diagnostic procedures and reporting [43].

### Construction of the histologically assigned spectral database: spectral processing, bioinformatics and statistical analysis

To ensure only samples with adequate spectra and representative of true pathological change were used to build the tissue-type MS database, strict inclusion and exclusion criteria were determined (detailed in Additional file 2: Table S2); 40 specimen data files were excluded from a total of 399, leaving 359 for analysis of normal (B1 and B2) versus tumour (B5a and B5b). Raw mass spectrometric data were processed with Offline Model Builder (OMB-v29, Waters Research Centre, Hungary) with a bin of 0.01 *m/z* with background subtraction and lock mass correction (699.497 *m/z*). Spectra acquired from all sample burns were averaged to produce a single mass spectrum per sample. The mass spectra from all samples were imported into Matlab 2014a (Mathworks, MA, USA) and profile mode alignment [44] was applied prior to peak picking. The data were median fold change normalised and log transformed prior to multivariate statistical analysis. Principal component analysis (PCA) was used to identify trends in the data. Linear discriminant analysis (LDA) was used to identify spectral differences between cancer and normal tissue. Classification performance was recorded for each model with a leave-one-patient-out cross-validation scheme.

### Lipid phenotyping of normal and cancerous human breast tissue with REIMS

A sub-set of high-density (90–100%) cancer samples (*n* = 17) was compared to randomly selected normal breast tissue (*n* = 17) to determine the differences in peak intensities between cancerous and normal tissue. Total intensity normalization was performed prior to univariate analysis. The Mann-Whitney *U* test was performed, with Benjamini-Hochburg-Yekutieli false discovery rate correction accounting for multiple testing (*p* < 0.05). Isotope peaks were excluded. Thawed frozen normal (*n* = 2) and cancerous (*n* = 2) breast tissues were sampled in *cut* mode for 10–15 seconds to allow fragmentation of ions and collection of tandem MS (MS/MS) data. Tentative ion identification was performed by searching peak *m/z* values in the METLIN metabolite database and with LIPID MAPS® online tools [45, 46], which was refined using the MS/MS data.

### Ex-vivo iKnife validation

**Fig. 1:**
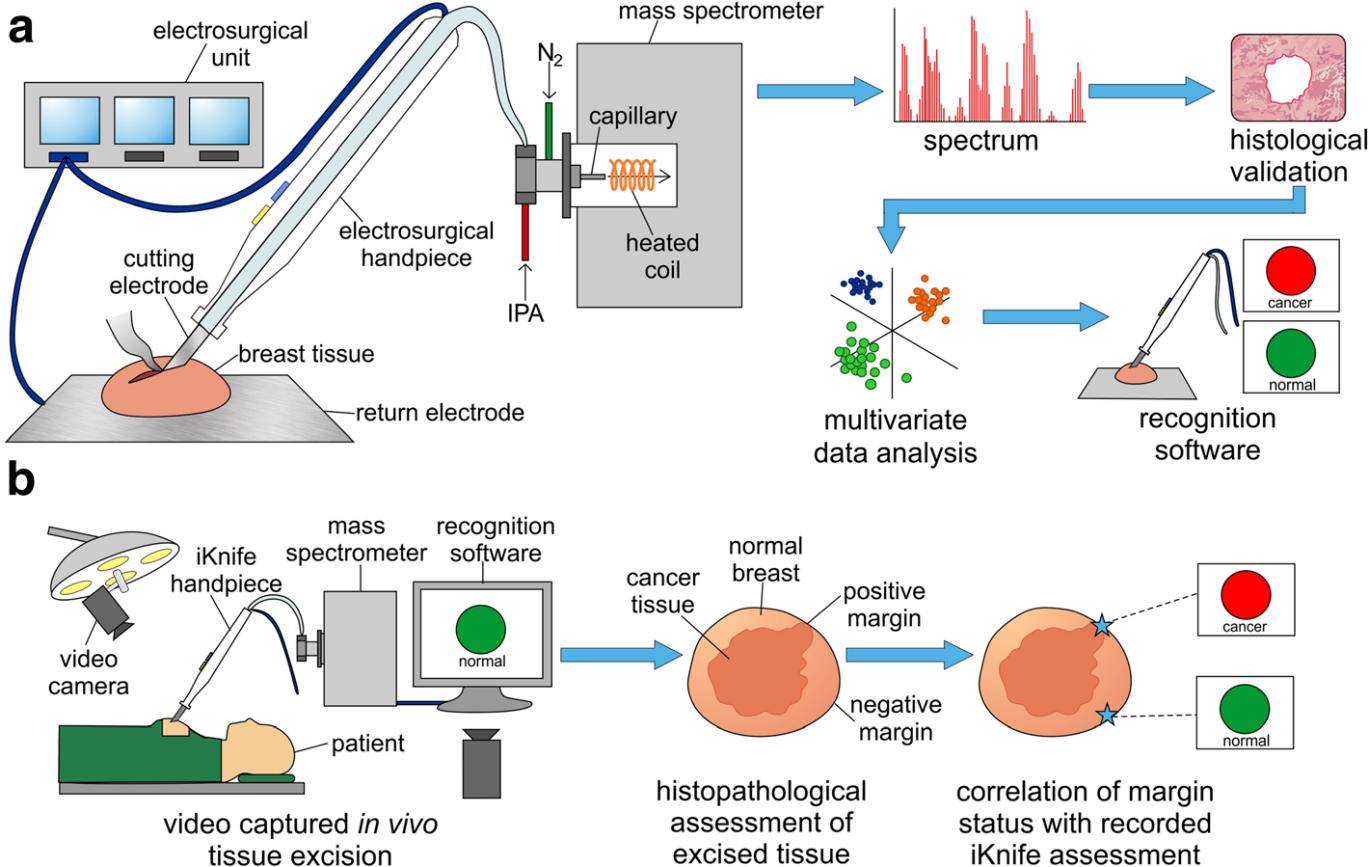
Ex-vivo and intraoperative workflows. **a** Ex-vivo workflow from generation of spectra by mass spectrometry (MS) analysis of surgical aerosol through to model building by multivariate statistics leading to ex-vivo recognition of tissue in real time. **b** Intraoperative workflow from generation of spectra in real time by on-line MS analysis, through to determination of margin status by histopathological assessment and correlation to iKnife results

The accuracy of the combined *cut* and *coag* ex-vivo model was tested by exporting the OMB statistical model data into purpose-built OMB recognition software (V29, Waters Research Centre, Hungary). The following parameters were set: mass range 600–1000 *m/z*, 0.1 bin, background subtraction on and lock mass correction to 699.497 *m/z* (phosphatidic acid (PA) (36:2)). REIMS analysis was performed in *cut* and *coag* modalities on new fresh and defrosted, normal and tumour breast tissues with both macro and microscopic histological agreement of tissue type. The validation spectra were pre-processed as previously described, then transformed to the linear discriminant space and classified to the closest class (i.e. normal or tumour) within the space using Mahalanobis squared distances. Recognition output was compared to histopathological results from H&E slides of the same tissue sample. For the correct classification of normal tissue, spectra from all sampling points within the specimen must have been registered as “normal” breast. Conversely, for tumour, at least one spectrum detected within a sample must have been considered positive for “tumour”. Figure 1a illustrates the ex-vivo recognition workflow. For additional validation, real-time ex-vivo tumour detection was performed on three mastectomy sections using electrosurgical dissection through normal breast into tumour tissue and back to normal breast tissue. The acquisition of REIMS spectra was synchronized with a video recording (GoPro, CA, USA) of the smoke capture as the tissue was continuously cut or coagulated with an electrosurgical blade. OMB recognition software was used to classify tissue content and this was compared to macroscopic tissue observations.

### Intraoperative iKnife - proof of principle

To determine if the ex-vivo method was applicable to the intraoperative environment we ran an intraoperative proof-of-principle study. A modified Xevo G2-XS mass spectrometer (Waters, UK) was installed in the operating theatre and a commercially available sterile (Surg-N-Vac or AccuVac, Covidien, UK) hand-piece was connected to the instrument. Aerosol produced as a result of electrosurgical tissue manipulation was continuously aspirated into the instrument throughout the operation. Video footage was simultaneously recorded, capturing all activities occurring at the operative scene (GoPro, CA, USA) in a time-synchronized manner with the acquisition of spectral data. Video recordings enabled retrospective orientation of spectral data with regard to three-dimensional margins. However, presently sample size is inadequate for interpretation of diagnostic accuracy. An optimised iKnife intraoperative workflow is displayed in Fig. 1b.

## Results

### REIMS spectral differences between tissue type and electrosurgical modality

**Fig. 2:**
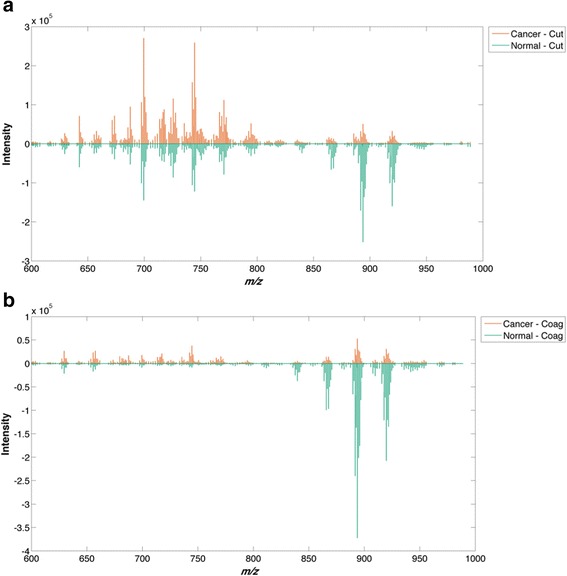
Mean spectral intensity for cancer and normal tissues during cutting (*cut*) and coagulation (*coag*) electrosurgical modalities. The *m*/*z* intensities are positive for both normal and tumour; here positive intensities are reflected opposite each other to illustrate similarities and differences between the groups. The intensity of triglycerides (850–1000 *m*/*z*) is greater than the intensity of phospholipids (600–850 *m*/*z*) in normal breast tissue (**a**, **b**), whilst the membrane phospholipids are more dominant in breast cancer (**a**, **b**). Differences are observed between *cut* mode (**a**) and *coag* mode (**b**). *Coag* mode, compared to *cut* mode, gives a higher triglyceride signal but lower phospholipid signal

Average spectra obtained from 253 normal (B1 and B2) and 106 tumour (B5a and B5b) samples were used to create typical REIMS multispectral “fingerprints” for normal and cancerous breast tissue in both *cut* and *coag* electrosurgical modalities (Fig. 2). Spectral feature intensities were significantly dependent on the tissue type and electrosurgical setting used. Normal tissue demonstrated high intensity spectra in the phospholipid range (600–850 *m/z*) and the triglyceride range (850–1000 *m/z*) using *cut* mode and predominantly in the triglyceride range using *coag* mode. Conversely, tumour tissue demonstrated an increase in the phospholipid range and a decrease in the triglyceride range in both *cut* and *coag* modalities.

### Creation of a REIMS ex-vivo breast tissue classification model

**Fig. 3:**
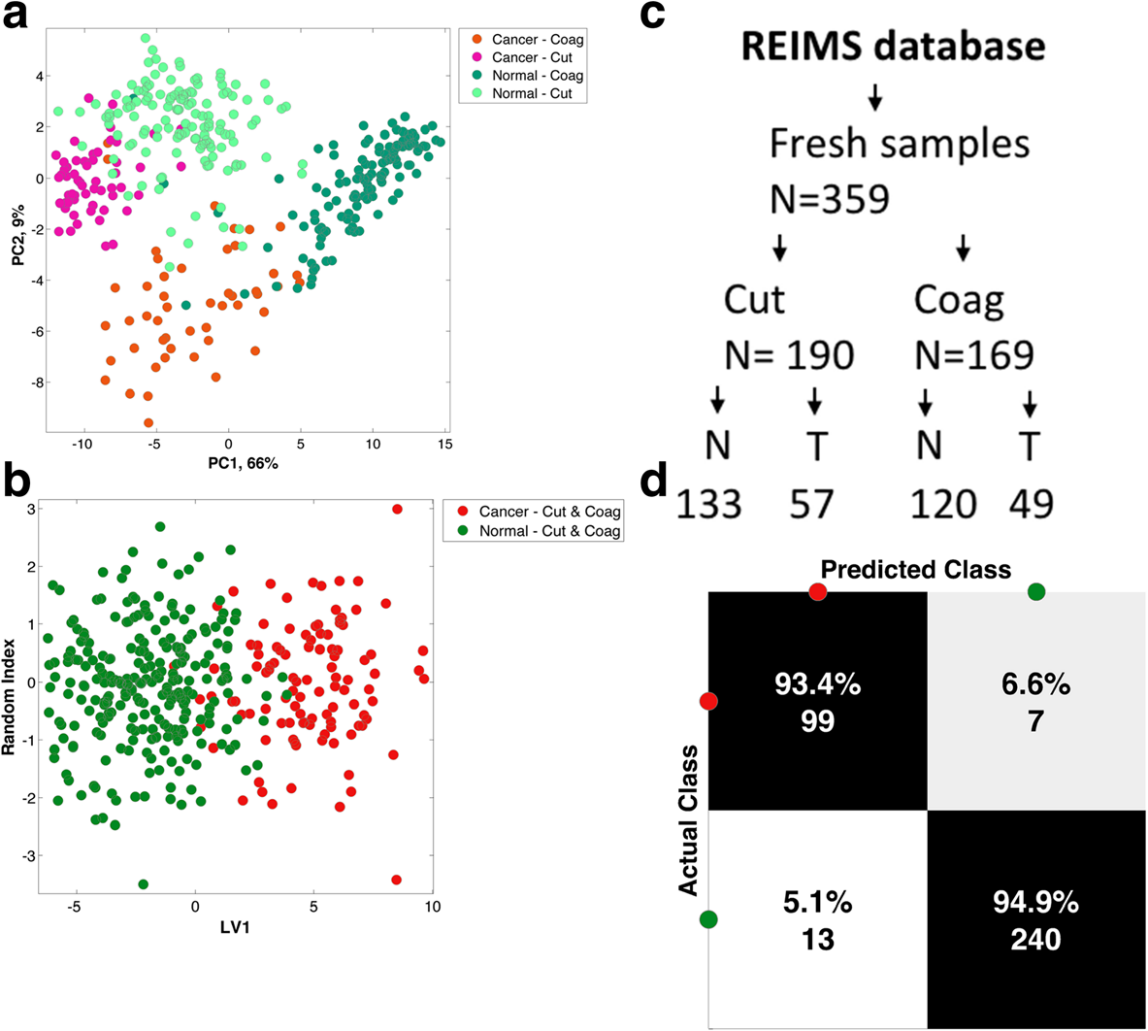
Multivariate statistical analysis of the combined *cut* and *coag* model. **a** Unsupervised principal component (*PC*) analysis of the spectral differences (600–1000 *m*/*z*) between normal tissue compared to breast cancer in the *cut* and *coag* electrosurgical modalities. **b** Supervised linear discriminant analysis plot comparing normal tissue (*N*) to tumour/cancer (*T*) regardless of electrosurgical modality. **c** Flow diagram of sample selection for building of the rapid evaporative ionisation mass spectrometry (*REIMS*) database. **d** Confusion matrix demonstrating diagnostic accuracy of the combined electrosurgical model following leave-one-patient-out cross-validation (LV1), with sensitivity (93.4%) and specificity (94.9%)

Statistical models were created for normal breast (B1 and B2) versus breast tumour (B5a and B5b) using *cut*, *coag* or a combination of the two (combined model). Data from 1158 sampling points from a total of 359 individual specimens; 253 normal (932 sampling points) and 106 tumour specimens (226 sampling points) from 113 patients were used to build the combined model (Table 1). The combined model was selected for recognition as both *cut* and *coag* modalities are frequently used interchangeably during breast cancer surgery. For the combined model, tumour class was distributed as 42 IDC, 8 ILC, 4 IMC and 2 DCIS (Table 1 and Additional file 1: Table S1). The mean (± standard deviation (SD)) age of patients in the combined group was 57.2 years (±13.48). The leave-one-patient-out cross-validation of the corresponding statistical model based on individual specimens (*n* = 359) resulted in overall accuracy of 94.4%, sensitivity of 93.4% and specificity of 94.9% (Fig. 3). For completeness, the *cut* model resulted in accuracy of 95.8%, sensitivity of 94.7% and specificity of 96.2% (Additional file 3: Figure S1), whilst the *coag* model resulted in overall accuracy of 94.7%, sensitivity of 93.9% and specificity of 95.0% (Additional file 4: Figure S2).

**Table 1:** Ex-vivo database model statistics and demographics

		*Cut*	*Coag*	*Cut* and *coag* combined
Total	Sampling points	634	524	1158
	Samples	190	169	359
	Patients	108	105	113
Normal (B1 and B2)	Sampling points	510	422	932
	Samples	133	120	253
	Patients	95	93	103
Tumour (B5a and B5b)	Sampling points	124	102	226
	Samples	57	49	106
	Patients	53	46	56
Tumour type	IDC	39	33	42
	ILC	8	7	8
	IMC	4	4	4
	DCIS	2	2	2
Tumour receptor status	ER+/HER2–negative	42	37	45
	ER+/HER2–positive	3	3	3
	ER–/HER2–positive	3	2	3
	Triple-negative	3	2	3
	DCIS	2	2	2
Age (mean)	All	57.56	57.16	57.15
	Normal	57.14	56.63	56.51
	Tumour	60.94	62.13	60.73
Model statistics	Sensitivity	94.7%	93.9%	93.4%
	Specificity	96.2%	95.0%	94.9%
	Accuracy	95.8%	94.7%	94.4%

Statistics and demographics displayed for three ex-vivo models (*Cut*, *Coag* and Combined). IDC invasive ductal carcinoma, *ILC* invasive lobular carcinoma, *IM*C invasive mucinous carcinoma, *DCIS* ductal carcinoma in situ, *ER+* oestrogen receptor positive, *ER*– oestrogen receptor negative, *HER2+* human epidermal growth factor receptor 2 positive, *HER*– human epidermal growth factor receptor 2 negative

### Identification of lipids in significant peaks by MS/MS

**Fig. 4:**
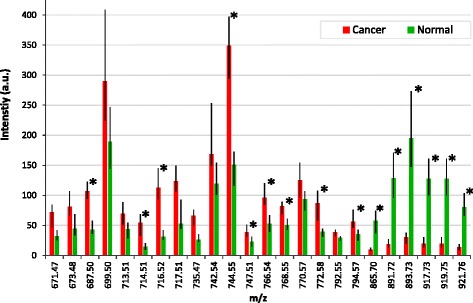
Graph shows statistically significant differences (*p* < 0.05) in the mean intensity of the *m*/*z* peaks in normal tissue and in cancer (*=q value (false discovery rate (FDR)-corrected *p* value) ≤0.001). *Range bars* represent the interquartile range. There were 18 peaks that increased in cancer within the phospholipid range (600–850 *m*/*z*); 6 peaks increased in normal tissue within the triglyceride range (850–1000 *m*/*z*). *a.u.* arbitrary units

**Fig. 5:**
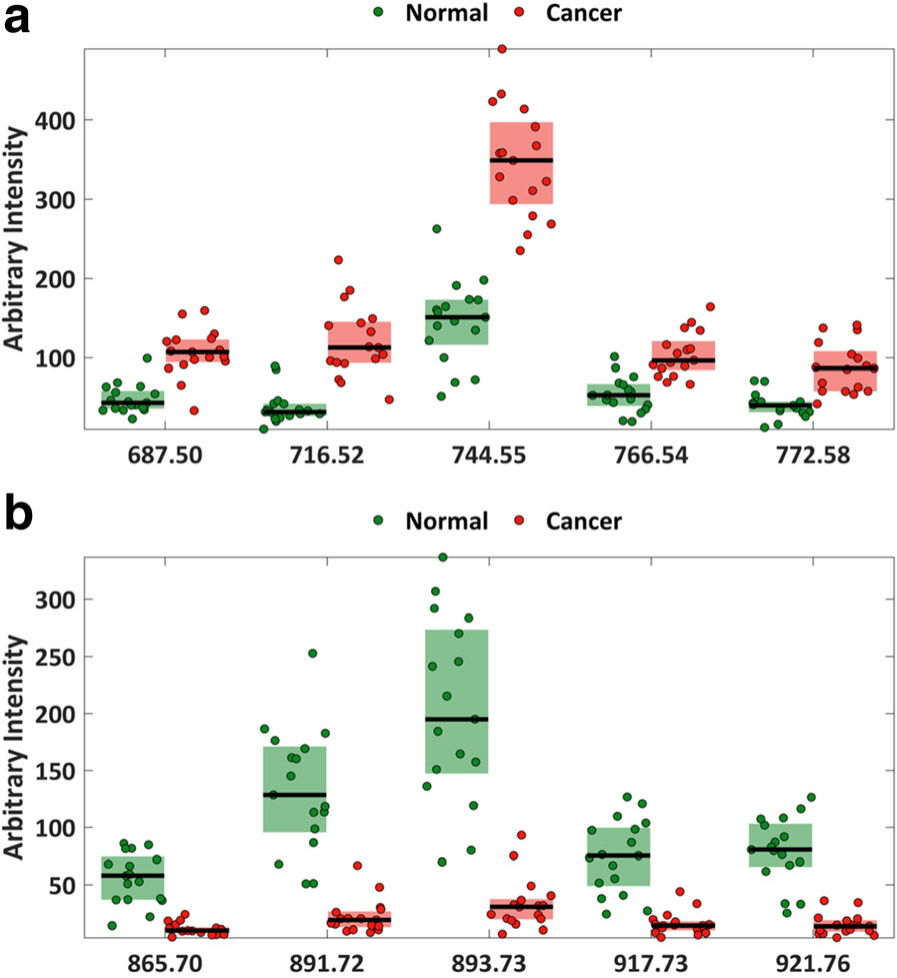
Intensities of five significant features that had higher intensities in cancer samples (**a**) and five significant features that had higher intensities in normal samples (**b**). The features are the most significant ones identified by univariate analysis (see Fig. 4 and Additional file 5: Table S3). The *bottom* and *top* of the *coloured band* represent, respectively, the 25th and 75th percentiles of the group, with the median denoted by the *black line*. The individual intensities for each group have been scattered with a random amount of *x-axis* positional variation

Univariate statistical analysis was used to identify significant peaks that differed between normal and cancerous tissue. After exclusion of isotopes, 24 significant peaks remained; 18 were higher in tumour tissue, with an average of 0.5 log_2_ fold increase compared to normal and 6 peaks were lower in tumour, with an average of 5.7 log_2_ fold decrease (Fig. 4 and Additional file 5: Table S3). The five most significant variables with higher cancer intensity are plotted in Fig. 5a, and the five variables with higher intensity in normal samples are plotted in Fig. 5b. MS/MS experiments were performed to identify the lipid species. Table 2 highlights the phospholipid species identified from the MS/MS spectra that were elevated in cancer compared to normal tissue (600–850 *m/z* range). All of the lipids identified in this range were glycerophospholipids with phosphatidylethanolamines (PEs) being the most commonly identified lipid species, followed by phosphatidylcholines (PCs), phosphatidic acids (PAs), phosphatidylserines (PSs) and phosphatidylglycerols (PGs) in that order. Table 3 summarises the triglyceride species identified from the MS/MS spectra of peaks that were significantly lower in cancer (850–1000 *m/z*).

**Table 2:** MS/MS-based phospholipid identification increased in breast cancer (600–850 *m*/*z*)

*m*/*z*	Lipid identification	Ion
671.47	PA (16:0/18:2)	[M-H]-
	PA (16:1/18:1)	[M-H]-
	PE (16:1/16:0)	[M-NH_3_-H]-
673.48	PA (16:0/18:1)	[M-H]-
	PA (18:0/16:1)	[M-H]-
	PE (16:0/16:0)	[M-NH_3_-H]-
687.5	PA (P-20:0/16:0)	[M-H]-
	PA (O-18:0/18:1)	[M-H]-
699.5	PE (16:0/18:1)	[M-NH_3_-H]-
	PA (18:1/18:1)	[M-H]-
	PA (18:0/18:2)	[M-H]-
713.51	PA (P-20:0/18:1)	[M-H]-
	PA (O-20:0/18:2)	[M-H]-
	PG (P-16:0/18:1)	[M-H_2_O-H]-
714.51	PE (16:0/18:2)	[M-H]-
	PE (16:1/18:1)	[M-H]-
	PE (16:2/18:0)	[M-H]-
716.52	PE (18:0/16:1)	[M-H]-
	PE (16:0/18:1)	[M-H]-
717.51	PA (O-16:0/20:4)	[M + Cl]-
	PC (16:0/16:0)	[M-CH_3_-H]-
735.47	PA (P-20:0/20:4)	[M-H]-
	PA (P-20:1/20:3)	[M-H]-
	PG (O-18:0/16:0)	[M-H]-
	PA (18:0/18:2)	[M + Cl]-
	PA (18:1/18:1)	[M + Cl]-
	PA (P-18:0/22:4)	[M-H]-
742.54	PE (18:0/18:2)	[M-H]-
	PE (18:1/18:1)	[M-H]-
	PC (16:1/18:1)	[M-CH_3_-H]-
	PC (16:0/18:2)	[M-CH_3_-H]-
744.55	PC (16:0/18:1)	[M-CH_3_-H]-
	PC (18:0/16:1)	[M-CH_3_-H]-
	PE (18:0/18:1)	[M-H]-
747.51	PA (18:1/22:5)	[M-H]-
	PA (18:2/22:4)	[M-H]-
	PE (18:1/20:4)	[M-NH_3_-H]-
	PE (16:0/22:5)	[M-NH_3_-H]-
	PE (18:0/20:5)	[M-NH_3_-H]-
766.54	PS (P-16:0/20:4)	[M-H]-
	PE (18:0/20:4)	[M-H]-
	PC (16:0/20:4)	[M-CH_3_-H]-
768.55	PC (18:1/18:2)	[M-CH_3_-H]-
	PC (16:0/20:3)	[M-CH_3_-H]-
	PE (20:2/18:1)	[M-H]-
	PE (18:0/20:3)	[M-H]-
770.57	PE (18:0/20:2)	[M-H]-
	PC (18:1/18:1)	[M-CH_3_-H]-
	PC (18:0/18:2)	[M-CH_3_-H]-
772.58	PE (18:0/20:1)	[M-H]-
	PC (18:0/18:1)	[M-CH_3_-H]-
	PS (O-18:0/18:2)	[M-H]-
	PE (P-18:1/22:6)	[M-H]-
792.55	PC (16:0/18:2)	[M + Cl]-
	PC (18:2/20:3)	[M-CH_3_-H]-
	PC (18:0/20:5)	[M-CH_3_-H]-
	PC (18:1/20:4)	[M-CH_3_-H]-
	PE (18:1/22:4)	[M-H]-
	PE (18:0/22:5)	[M-H]-
794.57	PC (16:0/22:4)	[M-CH_3_-H]-
	PC (18:1/20:3)	[M-CH_3_-H]-
	PC (18:0/20:4)	[M-CH_3_-H]-
	PC (16:0/18:1)	[M + Cl]-

Possible lipid identifications from tandem mass spectrometry (MS/MS) data for each significant *m*/*z* peak in negative mode. Numbers within brackets represent the number of carbons in the fatty acid chain, followed by the number of double bonds (C:N). *PA* phosphatidic acid, *PE* phosphatidylethanolamine, *PC* phosphatidylcholine, *PS* phosphatidylserine

**Table 3:** Triglyceride identification increased in normal breast tissue (850–1000 *m*/*z*)

*m*/*z*	Lipid identification	Ion
865.70	TG (50:2)	[M + Cl]-
891.72	TG (52:3)	[M + Cl]-
893.73	TG (52:2)	[M + Cl]-
917.73	TG (54:4)	[M + Cl]-
919.75	TG (54:3)	[M + Cl]-
921.76	TG (54:2)	[M + Cl]-

Possible triglyceride species identified in the mass spectrometry spectra of *m*/*z* values that are significantly lower in tumour tissue. Triglycerides are denoted as TG (C:N) where C corresponds to the sum of the carbon atoms in the three fatty acid chains and N corresponds to the sum of the double bonds in the fatty acid chains

### Ex-vivo validation of iKnife

**Fig. 6:**
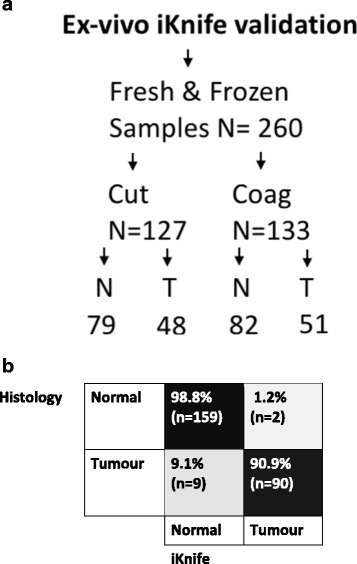
Ex-vivo validation of recognition software with new samples. **a** Flow diagram of samples used in the ex-vivo validation experiment. *N* normal tissue, T tumour tissue. **b** Confusion matrix demonstrating diagnostic accuracy of the combined electrosurgical model with a validation set of new fresh and frozen tissues. On analysis of diagnostic accuracy, sensitivity was 90.9% and specificity 98.8%

**Fig. 7:**
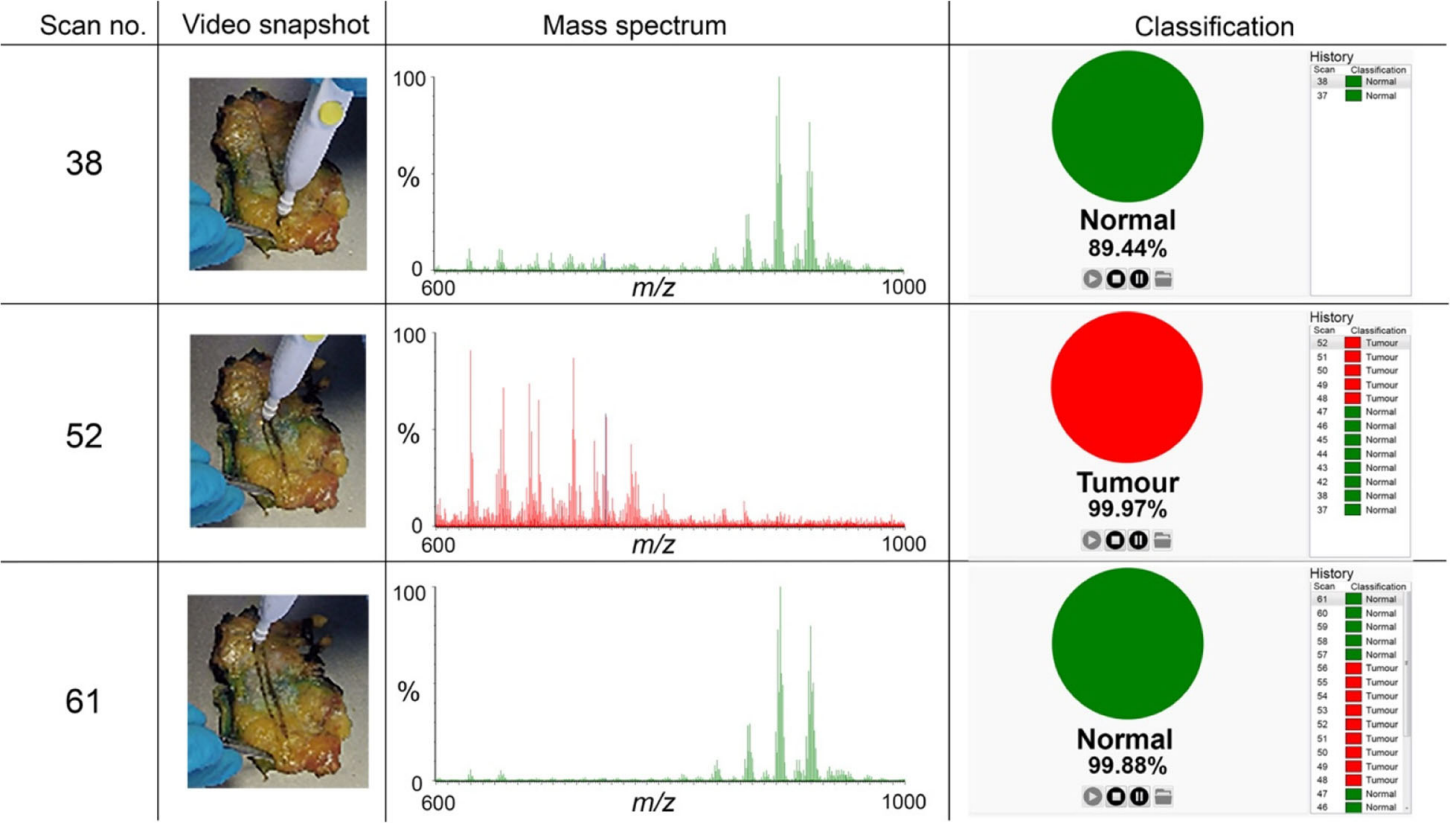
Ex-vivo validation case study. An electrosurgical hand-piece was moved through the mastectomy specimen in *coag* mode from normal breast tissue, into tumour and out through normal tissue. A simultaneous video recording reveals the position of the hand-piece in relation to the specimen and the generated spectra and demonstrates good correlation with the recognition software compared to macroscopic findings

New frozen and fresh breast specimens (*n* = 260) not previously used for ex-vivo model creation were subjected to iKnife analysis using both *cut* and *coag* modalities, employing an identical ex-vivo methodology combined with interpretation by bespoke iKnife recognition software (OMB V29). For the *cut* modality there were 79 normal and 48 tumour specimens and for the *coag* modality there were 82 normal and 51 tumour samples. Recognition software was concordant with final histopathological assessment in 249 out of 260 specimens, producing an overall model accuracy of 95.8% with a sensitivity of 90.9% and specificity of 98.8% (Fig. 6). Finally, the iKnife was used to perform a continuous dissection line through the tissue in *cut* and *coag* mode from normal, through tumour returning to normal tissue, in a case series of three whole mastectomy slices with large tumours (>3 cm). OMB recognition results demonstrated good overall classification (224/231 spectra, 97% accuracy) with video synchronised macroscopic tissue correlation (Fig. 7).

### Intraoperative iKnife - proof of principle

**Fig. 8:**
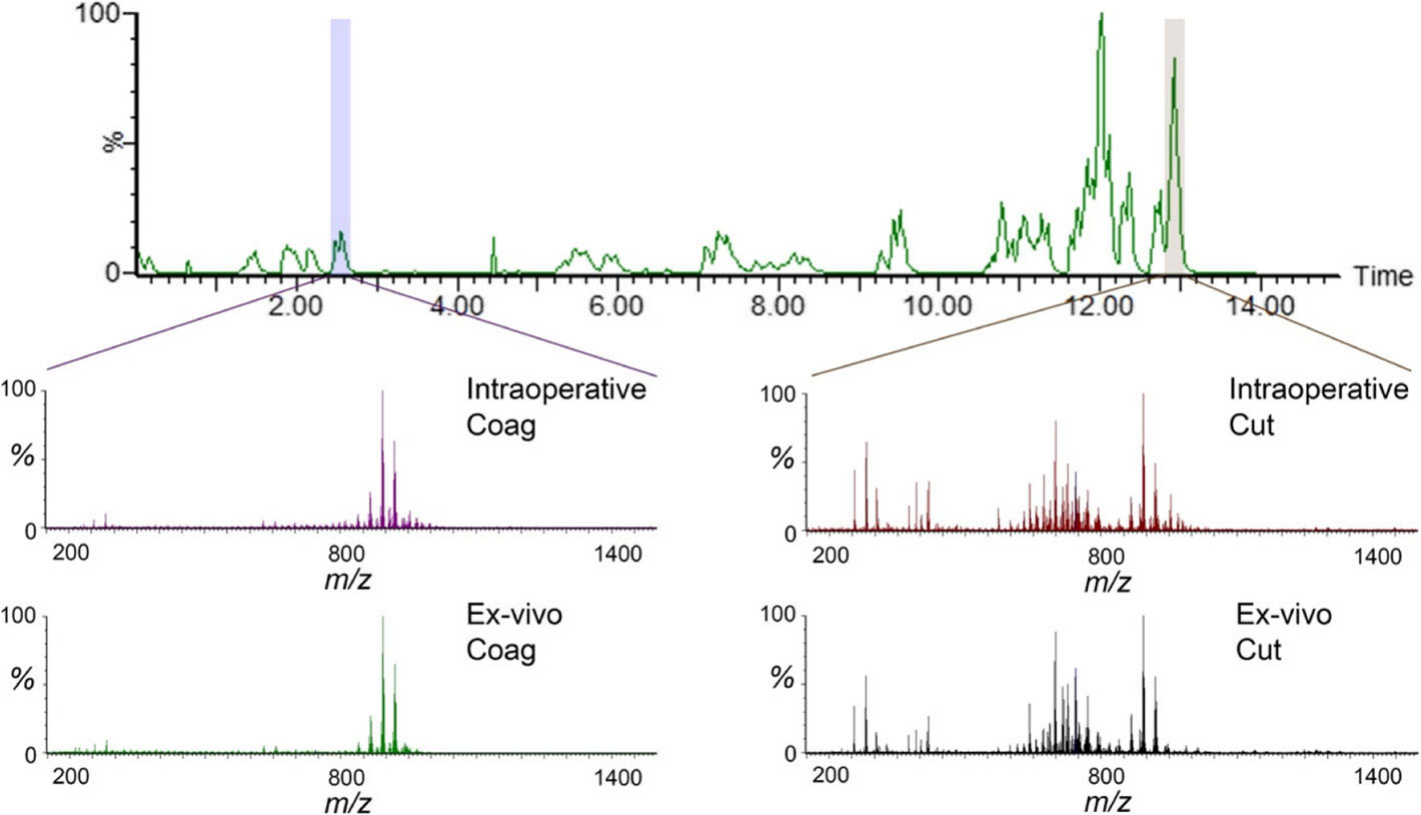
Collection of intraoperative mass spectral data with comparison to ex-vivo spectra. Spectral intensity over time obtained throughout entire surgery (14 minutes), one spectra obtained per second. Intraoperative spectral differences highlighted in *cut* (*right*) and *coag* (*left*) modalities observed in normal tissue and compared to similar spectra observed in two ex-vivo examples

The iKnife was used during the entire surgical intervention in six case studies as a proof of principle of the intraoperative method. The mean time from electrosurgical activation to detection of online analysis was 1.80 seconds (SD ±0.40). High intensity spectra (total ion count (TIC) = e7-e8) were obtained in all surgical interventions both in *cut* and *coag* mode throughout the operation and were comparable in intensity (TIC = e6-e8) and overall visual morphology to the ex-vivo spectra (Fig. 8). Of the intraoperative spectra across six surgical interventions, 99.27% (*n* = 5422/5462) were interpretable by the ex-vivo model. Only 0.73% (*n* = 40/5462) of spectra were classified as outliers (greater than 2 SD) according to the ex-vivo classification model.

## Discussion

A REIMS-based histological identification method has been successfully optimised for the real-time analysis of heterogeneous breast tissues. The construction of an ex-vivo database from fresh breast tissues demonstrated significant differences in spectra related to disparity in lipid metabolism between normal breast tissues and breast cancer. Spectral differences observed between *cut* and *coag* electrosurgical modalities have been combined to create a multivariate statistical model allowing the use of both modes interchangeably. Leave-one-patient-out cross-validation demonstrates this model can detect tumour with a sensitivity of 94.9% and exclude tumour with a specificity of 93.4%, results that rival other IMA techniques [34].

MS/MS has been used to characterise key lipid species present in normal and cancerous breast tissue. Abundance of ions associated with phospholipid species (600–850 *m*/*z*) were increased in cancer tissues compared to healthy tissue types, whereas intensity of ions associated with triglyceride species (850–1000 *m*/*z*) were decreased; a finding also reported by using alternative techniques [47, 48]. Phospholipids serve as chief components of biological membranes and hence they are indispensable for proliferating cells [49]. An increase in phospholipid synthesis is associated with lipogenic enzymes including fatty acid synthase and acetyl-CoA carboxylase a, which are commonly upregulated in breast cancer [50]. Lipid species, PE and PC, identified by REIMS MS/MS have also been identified by other studies to be increased in breast cancer cells [48]. Interestingly, PC (16:0/16:0), PC (18:0/20:4), PC (18:1/20:4) have been previously identified to be associated with ER-negative tumours and higher grade tumours, and associated with decreased overall survival in breast cancer patients [50].

Significant advances in software development have enabled real-time analysis of tissue composition and rapid comparison against the multispectral database of ex-vivo tissues. Specifically, we were clearly able to observe spectra obtained in real time with onscreen classification. Prospective ex-vivo validation with bespoke recognition software provides evidence that the statistical model is fit for purpose with high diagnostic accuracy demonstrated (90.9% sensitivity, 98.8% specificity) for both fresh and defrosted breast tissues.

Logistical barriers have been overcome and the iKnife has now been successfully introduced to the operating theatre, with a method that has been demonstrated as a proof of concept, to function well during breast surgery. Despite additional factors to consider such as blood flow and body temperature, high intensity mass spectral data were obtained during breast surgery, comparable to data obtained in the ex-vivo setting. An ex-vivo classification model was able to recognize a very high proportion of intraoperative spectra (>99%), which suggests that ex-vivo models trained on tissue type will be able to guide intra-operative analysis. Analysis of the diathermy smoke plume can be performed throughout the entire operation with results presented on screen within 2 seconds of diathermy activation. Hand-piece modifications could further improve the speed of results. Increased intraoperative numbers and appropriate translation of the ex-vivo recognition software for intra-operative use is required before conclusions can be made on the intraoperative diagnostic accuracy, and this remains the focus of our ongoing work.

The definition of what constitutes a positive margin continues to be debated; however, recent guidelines from the USA point to a narrowing of the accepted positive margin distance. The Society of Surgical Oncology and American Society for Radiation Oncology (SSO-ASTRO) advises that in invasive disease a negative margin should be considered as “no tumour on ink” [51]. This is based on the results from a meta-analysis that demonstrated an increase of 2.44 in the odds ratio of local recurrence for positive margins (tumour on ink) but with no significant benefits demonstrated for wider margins [8]. Interestingly, only relatively small further reductions in re-operation rates from 20.2% to 16.5% [52] and from 21.4% to 15.1% [53] have been observed following acceptance of the 2014 SSO-ASTRO guidelines and these reduced rates could still be considered excessive. Extrapolation of re-operative rates of 15% combined with the annual incidence of breast cancer and the increasing popularity of breast conserving surgery, amounts to many thousands of women undergoing potentially unnecessary operations with a significant health and economic cost to the patient and healthcare provider [13]. The fact that re-operation rates of 3.6% have been demonstrated following breast conserving surgery with the use of routine frozen section margin assessment [17] provides evidence that IMA techniques or technologies with high diagnostic accuracy could be expected to substantially reduce positive margin rates beyond those achieved by a reduction in positive margin width alone. It is therefore our opinion that there continues to be a need for accurate and rapid IMA technology capable of further reducing re-operation rates.

Although there are numerous established and emerging IMA devices, penetration to routine practice has been poor. Pathological techniques require complicated logistics between the operating theatre and pathology departments, sufficiently trained pathologists are a scarce resource and time taken to report results can be long and can delay operative workflow (24–50 minutes) [54, 55]. Both SR and IOUS guided surgery can be used directly by surgeons within the operating theatre; however neither technique is as accurate as pathological approaches and both are subject to intraobserver error [34]. A common limitation of all emerging technologies such as MarginProbe™ [23, 24], ClearEdge™ [25] or OCT [30] is the need to disrupt workflow demanding an additional probe during resection or specimen analysis following resection. Following excision, the exact orientation of the surgical specimen can prove to be challenging and this may affect accurate margin identification.

As a margin detection and optimisation device, the REIMS-based iKnife has a significant advantage in that there is no disruption to standard oncological workflow because the margin control device is coupled to the resection tool. Furthermore, results may be obtained fast enough to alter tissue excision in real time and hence may reduce problems with retrospective tissue orientation. Perhaps the most exciting attribute of the iKnife is the ability to compare chemical changes in cellular metabolism with tissue morphology as determined by pathological assessment. The iKnife is envisaged to be more than just an IMA tool and has the potential to provide real time chemical information about individual tumour biology, important in an era of precision medicine as we move towards offering bespoke treatments based on tumour/tissue biology [56].

### Limitations

Due to the width of the electrosurgical blade (4 mm) the iKnife has relatively low resolution that may lead to dilution of tumour cellular content by normal cells, which may provoke a false positive result. REIMS is a destructive process, therefore it is impossible to be certain of the histology of the exact cells under analysis. The need to perform REIMS analysis on tissue prior to formalin fixation and microscopic assessment limited the number of cases of ductal carcinoma in situ (B5a) that were available for inclusion. There is uncertainty about how the iKnife will classify solid benign lesions. In a sub-set analysis of tumour (B5b) versus fibroadenoma (B2) (Additional file 6: Figure S3), good sensitivity (94.1%) but poorer specificity (87.3%) was observed, indicating that the spectral differences between these two groups are subtler than comparisons between either of these entities and normal tissue.

## Conclusion

A mass spectrometric method for the rapid analysis of heterogeneous breast tissues has been developed. REIMS analysis can be performed in both the *cut* and *coag* modes, which allows the surgeon to alternate between modes as clinically necessary. Preliminary data suggest the iKnife is capable of accurately separating breast tissue types by interpretation of the cellular chemical constituents. Recognition software enables real-time analysis of both ex-vivo and in-vivo breast tissue. The iKnife method has been optimised and further work will focus on determining the accuracy of the tool for intraoperative classification of resection margins.

## Additional files

Additional file 1: Table S1. Ex-vivo database tumour characteristics for samples included in the ex-vivo database. *ER* oestrogen receptor, *PR* progesterone receptor, *HER2* human epidermal growth factor receptor 2 (DOCX 156 kb).

Additional file 2: Table S2. Inclusion and exclusion criteria for construction of the histologically assigned spectral database; 40 specimen files were excluded from a total of 399, leaving 359 specimen files for analysis of normal tissue (B1 and B2) versus tumour (B5a and B5b) (DOCX 44 kb).

Additional file 3: Figure S1. Multivariate statistical analysis of the *cut* model. **a** Unsupervised principal component analysis (PCA) analysis of the spectral differences (600–1000 *m*/*z*) between normal tissue compared to breast cancer using the *cut* electrosurgical modality. **b** Supervised linear discriminant analysis (LDA) plot comparing normal tissue to tumour using *cut* mode. **c** Confusion matrix demonstrating diagnostic accuracy of the *cut* model, following leave-one-patient-out cross-validation, with sensitivity (94.7%) and specificity (96.2%) (DOCX 367 kb).

Additional file 4: Figure S2. Multivariate statistical analysis of the *coag* model. **a** Unsupervised PCA analysis of the spectral differences (600–1000 *m*/*z*) between normal tissue compared to breast cancer using *coag* electrosurgical modality. **b** Supervised LDA plot comparing normal tissue to tumour using *coag* mode. **c** Confusion matrix demonstrating diagnostic accuracy of the *coag* model following leave-one-patient-out cross-validation, with sensitivity (93.9%) and specificity (95.0%) (DOCX 332 kb).

Additional file 5: Table S3. Significant *m*/*z* peak differences in mean intensity between normal tissue and cancer, with fold changes and *q* value (false discovery rate (FDR)-corrected *p* value). Negative fold changes denote peaks that are lower in tumour compared with normal tissue (DOCX 48 kb).

Additional file 6: Figure S3. Multivariate statistical analysis of fibroadenoma (B2) compared to cancer (B5b). **a** Unsupervised PCA analysis of the spectral differences (600–1000 *m*/*z*) between fibroadenoma samples compared to breast cancer using combined *cut* and *coag* electrosurgical modalities. **b** Supervised LDA plot comparing fibroadenoma to cancer using *cut* and *coag* modes. **c** Confusion matrix demonstrating diagnostic accuracy of the model: 55 solid fibroadenoma samples are compared to 101 tumour (B5b) samples. Sensitivity of tumour classification is high at 94.1% but specificity for the diagnosis of benign fibroadenoma is lower at 87.3% (DOCX 364 kb).

## Abbreviations

BCS: Breast conserving surgery
DCIS: Ductal carcinoma in situ
DESI: Desorption electrospray ionisation
ER: Oestrogen receptor
H&E: Haematoxylin and eosin
HER2: Human epidermal growth factor receptor 2
IBTR: Ipsilateral breast tumour regional recurrence
IDC: Invasive ductal carcinoma
iKnife: Intelligent knife
ILC: Invasive lobular carcinoma
IMA: Intraoperative margin assessment
IMC: Invasive mucinous carcinoma
IOUS: Intraoperative ultrasound
IPA: Isopropyl alcohol / propan-2-ol
LDA: Linear discriminant analysis
MALDI: Matrix-assisted laser desorption ionisation
MS/MS: Tandem mass spectrometry
MS: Mass spectrometry
OCT: Optical coherence tomography
OMB: Offline model builder
PAs: Phosphatidic acids
PCA: Principal component analysis
PCs: Phosphatidylcholines
PEs: Phosphatidylethanolamines
PGs: Phosphatidylglycerols
PR: Progesterone receptor
PSs: Phosphatidylserines
REIMS: Rapid evaporative ionisation mass spectrometry
RF: Radiofrequency
SD: Standard deviation
SR: Specimen radiology
TG: Triglycerides
TIC: Total ion count
UK: United Kingdom
USA: United States of America
