# Supplementary material for: Rapid evaporative ionisation mass spectrometry of electrosurgical vapours for the identification of breast pathology: towards an intelligent knife for breast cancer surgery
Source: Breast Cancer Res. 2017 May 23;19:59. doi: 10.1186/s13058-017-0845-2 (PMC5442854; doi:10.1186/s13058-017-0845-2)
Supplement: Supplementary file 1 — Ex-vivo database tumour characteristics for samples included in the ex-vivo database. ER oestrogen receptor, PR progesterone receptor, HER2 human epidermal growth factor receptor 2 (DOCX 156 kb). [file 13058_2017_845_MOESM1_ESM.docx]

| **Sample ID** | **Age** | ***Histology*** | **Type** | **Neoadjuvant** | **Grade** | **ER** | **PR** | **HER2** |  |
| --- | --- | --- | --- | --- | --- | --- | --- | --- | --- |
| 134 | 78 | Tumour | IDC | No | 2 | 8 | 0 | 0 | **ER+/HER2-ve** |
| 135 | 60 | Tumour | IDC | No | 3 | 8 | 5 | 0 | **ER+/HER2-ve** |
| 145 | 50 | Tumour | IDC | No | 1 | 7 | 7 | 0 | **ER+/HER2-ve** |
| 160 | 44 | Tumour | IDC | No | 2 | 8 | 8 | 0 | **ER+/HER2-ve** |
| 161 | 66 | Tumour | ILC | No | 2 | 8 | 8 | 0 | **ER+/HER2-ve** |
| 179 | 86 | Tumour | IMC | No | 2 | 8 | 7 | 0 | **ER+/HER2-ve** |
| 180 | 66 | Tumour | IDC | Letrozole | 3 | 8 | 5 | 0 | **ER+/HER2-ve** |
| 191 | 81 | Tumour | ILC | No | 1 | 8 | 8 | 0 | **ER+/HER2-ve** |
| 193 | 73 | Tumour | IMC | No | 2 | 8 | 5 | 0 | **ER+/HER2-ve** |
| 198 | 43 | Tumour | IDC | No | 2 | 8 | 8 | 0 | **ER+/HER2-ve** |
| 223 | 34 | Tumour | IDC | No | 2 | 7 | 8 | 0 | **ER+/HER2-ve** |
| 231 | 50 | Tumour | IDC | No | 1 | 8 | 6 | 0 | **ER+/HER2-ve** |
| 233 | 50 | Tumour | IDC | No | 2 | 0 | 0 | 3 | **ER-/HER2+ve** |
| 242 | 56 | Tumour | IDC | No | 2 | 7 | 3 | 0 | **ER+/HER2-ve** |
| 245 | 50 | Tumour | ILC | No | 2 | 8 | 8 | 0 | **ER+/HER2-ve** |
| 246 | 51 | Tumour | IDC | No | 2 | 7 | 6 | 0 | **ER+/HER2-ve** |
| 247 | 63 | Tumour | IDC | No | 2 | 8 | 8 | 0 | **ER+/HER2-ve** |
| 271 | 72 | Tumour | ILC | No | 2 | 8 | 3 | 0 | **ER+/HER2-ve** |
| 272 | 60 | Tumour | IDC | No | 2 | 2 | 0 | 1 | **Triple neg** |
| 283 | 53 | Tumour | IDC | Letrozole | 2 | 8 | 6 | 0 | **ER+/HER2-ve** |
| 285 | 74 | Tumour | IMC | No | 3 | 8 | 8 | 2 (-ve) | **ER+/HER2-ve** |
| 287 | 88 | Tumour | IDC | No | 1 | 8 | 8 | 0 | **ER+/HER2-ve** |
| 288 | 57 | Tumour | IDC | No | 1 | 8 | 0 | 0 | **ER+/HER2-ve** |
| 289 | 55 | Tumour | ILC | No | 2 | 7 | 7 | 0 | **ER+/HER2-ve** |
| 293 | 67 | Tumour | IDC | No | 3 | 8 | 8 | 0 | **ER+/HER2-ve** |
| 294 | 41 | Tumour | IDC | FEC+Taxotere | 2 | 8 | 4 | 1 | **ER+/HER2-ve** |
| 296 | 41 | Tumour | IDC | No | 2 | 8 | 5 | 0 | **ER+/HER2-ve** |
| 310 | 39 | Tumour | IDC | No | 1 | 6 | 6 | 0 | **ER+/HER2-ve** |
| 315 | 42 | Tumour | IDC | No | 3 | 6 | 4 | 0 | **ER+/HER2-ve** |
| 316 | 67 | Tumour | IDC | No | 2 | 8 | 8 | 0 | **ER+/HER2-ve** |
| 323 | 72 | Tumour | IDC | Letrozole | 3 | 8 | 6 | 2 (-ve) | **ER+/HER2-ve** |
| 324 | 58 | Tumour | IDC | No | 2 | 8 | 8 | 0 | **ER+/HER2-ve** |
| 329 | 81 | Tumour | IDC | No | 3 | 8 | 0 | 3+ | **ER+/HER2+ve** |
| 330 | 54 | Tumour | ILC | No | 2 | 8 | 0 | 3+ | **ER+/HER2+ve** |
| 332 | 87 | Tumour | IDC | No | 2 | 8 | 8 | 0 | **ER+/HER2-ve** |
| 334 | 48 | Tumour | ILC | No | 2 | 8 | 8 | 0 | **ER+/HER2-ve** |
| 335 | 40 | Tumour | IDC | FEC | 2 | 8 | 8 | 2 (-ve) | **ER+/HER2-ve** |
| 339 | 76 | Tumour | IDC | Letrozole | 2 | 8 | 8 | 0 | **ER+/HER2-ve** |
| 340 | 46 | Tumour | IMC | No | 2 | 7 | 3 | 1 | **ER+/HER2-ve** |
| 342 | 52 | DCIS | DCIS | No | High/Intermediate |  |  |  | **DCIS** |
| 345 | 33 | Tumour | IDC | No | 3 | 8 | 5 | 0 | **ER+/HER2-ve** |
| 353 | 83 | Tumour | IDC | No | 2 | 0 | 0 | 0 | **Triple neg** |
| 357 | 78 | Tumour | IDC | No | 3 | 0 | 0 | 3+ | **ER-/HER2+ve** |
| 362 | 47 | Tumour | IDC | No | 1 | 8 | 8 | 0 | **ER+/HER2-ve** |
| 363 | 79 | Tumour | IDC | No | 1 | 8 | 8 | 0 | **ER+/HER2-ve** |
| 367 | 73 | Tumour | IDC | No | 3 | 0 | 0 | 0 | **Triple neg** |
| 373 | 47 | Tumour | IDC | No | 2 | 6 | 8 | 3+ | **ER+/HER2+ve** |
| 382 | 81 | Tumour | IDC | No | 2 | 8 | 5 | 1 | **ER+/HER2-ve** |
| 401 | 67 | Tumour | IDC | No | 2 | 8 | 6 | 2 (-ve) | **ER+/HER2-ve** |
| 410 | 68 | Tumour | ILC | No | 2 | 8 | 7 | 0 | **ER+/HER2-ve** |
| 442 | 68 | DCIS | DCIS | No | High |  |  |  | **DCIS** |
| 493 | 53 | Tumour | IDC | Letrozole | 2 | 8 | 5 | 0 | **ER+/HER2-ve** |
| 512 | 55 | Tumour | IDC | YES | 3 | 0 | 0 | 0 | **ER+/HER2-ve** |
| 514 | 65 | Tumour | IDC | No | 1 | 8 | 7 | 0 | **ER+/HER2-ve** |
| 536 | 64 | Tumour | IDC | No | 1 | 8 | 7 | 0 | **ER+/HER2-ve** |
| 540 | 69 | Tumour | IDC | No | 3 | 0 | 0 | 3+ | **ER-/HER2+ve** |
