# Supplementary material for: Rapid evaporative ionisation mass spectrometry of electrosurgical vapours for the identification of breast pathology: towards an intelligent knife for breast cancer surgery
Source: Breast Cancer Res. 2017 May 23;19:59. doi: 10.1186/s13058-017-0845-2 (PMC5442854; doi:10.1186/s13058-017-0845-2)
Supplement: Supplementary file 2 — Inclusion and exclusion criteria for construction of the histologically assigned spectral database; 40 specimen files were excluded from a total of 399, leaving 359 specimen files for analysis of normal tissue (B1 and B2) versus tumour (B5a and B5b) (DOCX 44 kb). [file 13058_2017_845_MOESM2_ESM.docx]

| **Inclusion Criteria** | **Exclusion Criteria** |
| --- | --- |
| breast tissue with definitive validated histopathological interpretation for normal, benign or cancer (i.e. B1, B2, B5a and B5b) | core biopsy specimens |
|  | lymph nodes |
| REIMS analysis using ForceTriad^TM^ in *Cut* or *Coag* pure mode | positive ion mode |
|  | poor quality mass spectra (i.e. no spectra, very low intensity, high baseline or excessively noisy spectra) |
| mass spectrometric analysis in negative ion mode | tumour present in less than 5% of sample slides |
|  | fibroadenoma |
