# Supplementary figures and images for: Rapid evaporative ionisation mass spectrometry of electrosurgical vapours for the identification of breast pathology: towards an intelligent knife for breast cancer surgery
Source: Breast Cancer Res. 2017 May 23;19:59. doi: 10.1186/s13058-017-0845-2 (PMC5442854; doi:10.1186/s13058-017-0845-2)

**
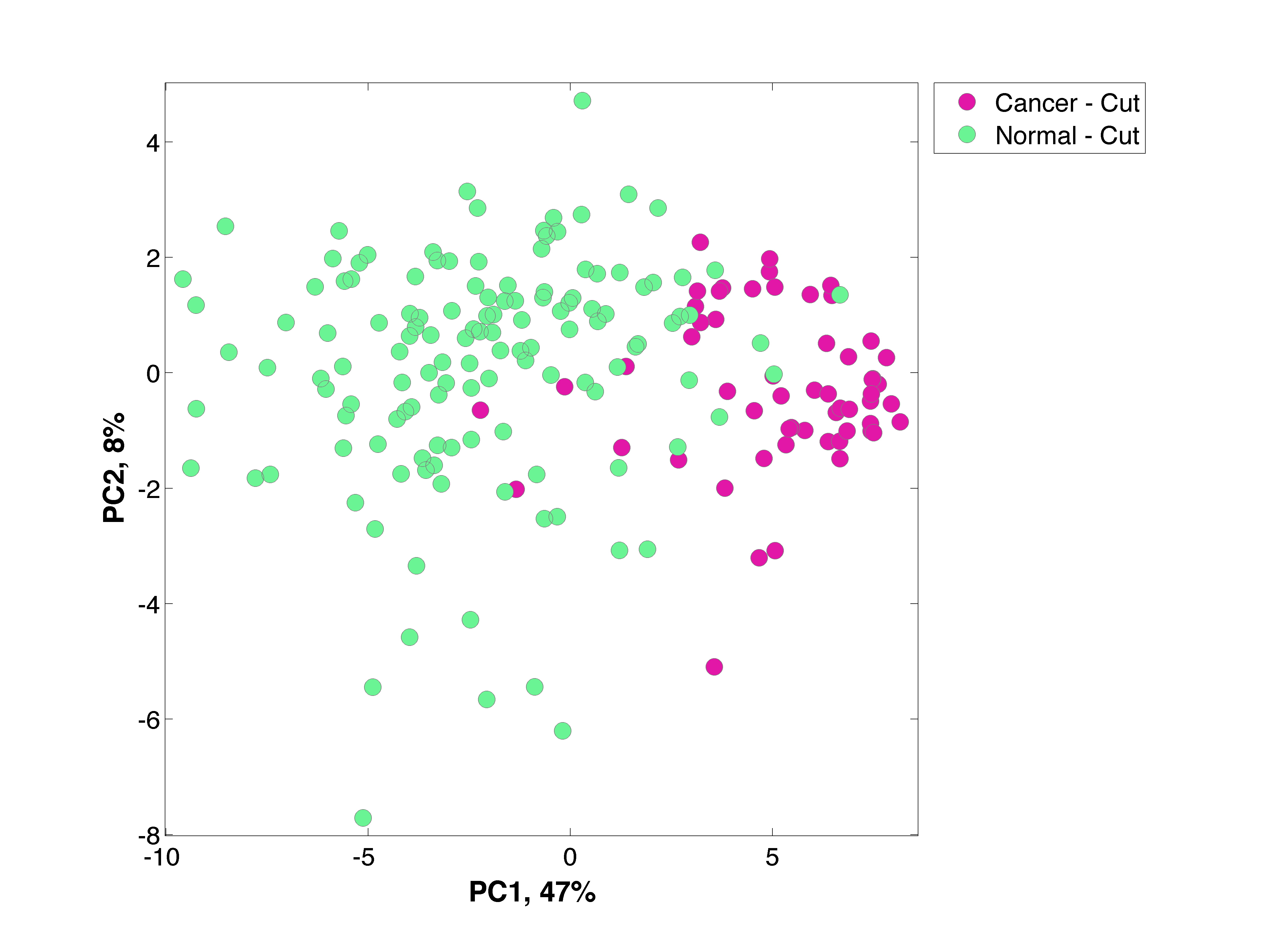
**

**a)**


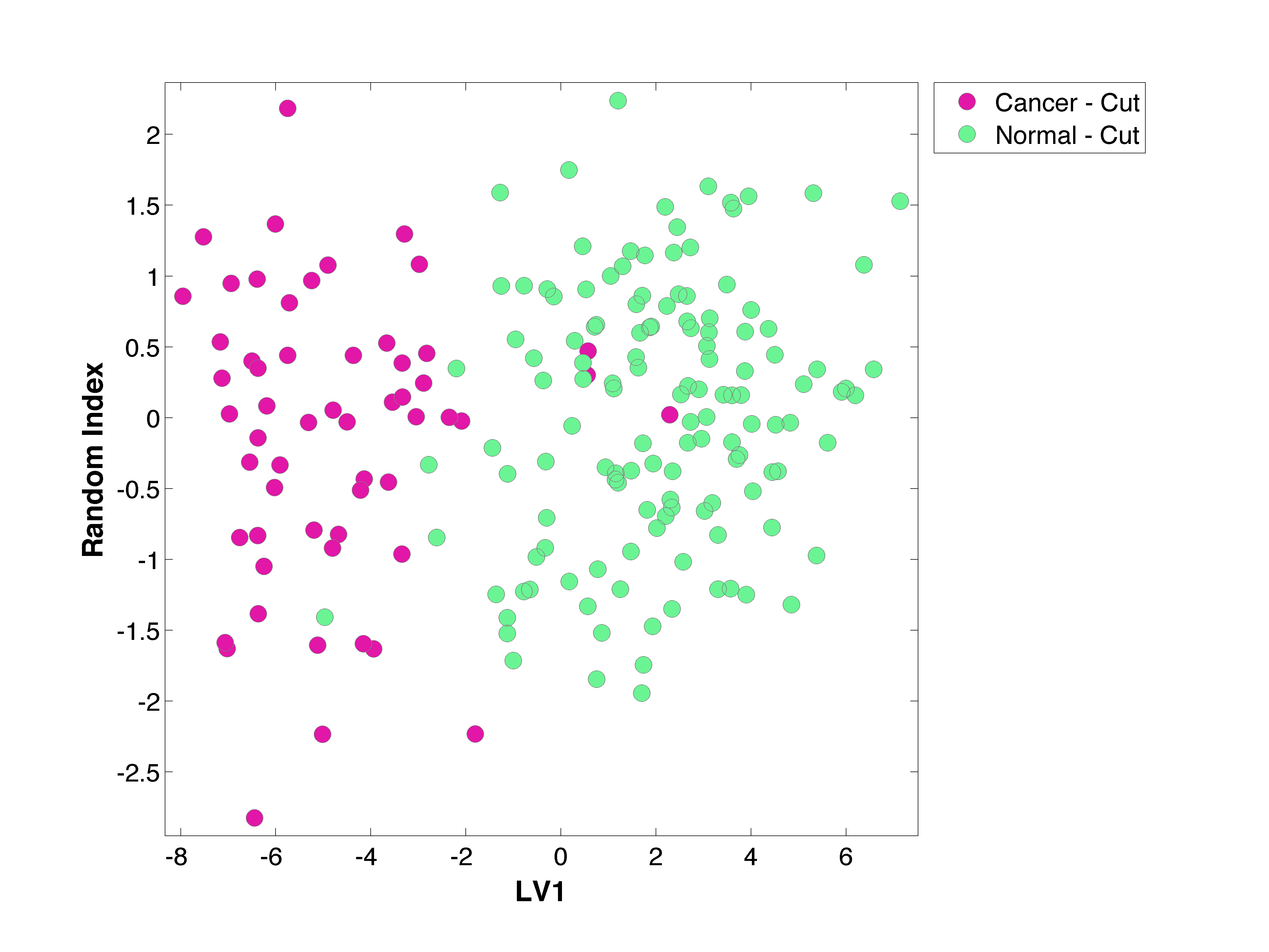


**b)**


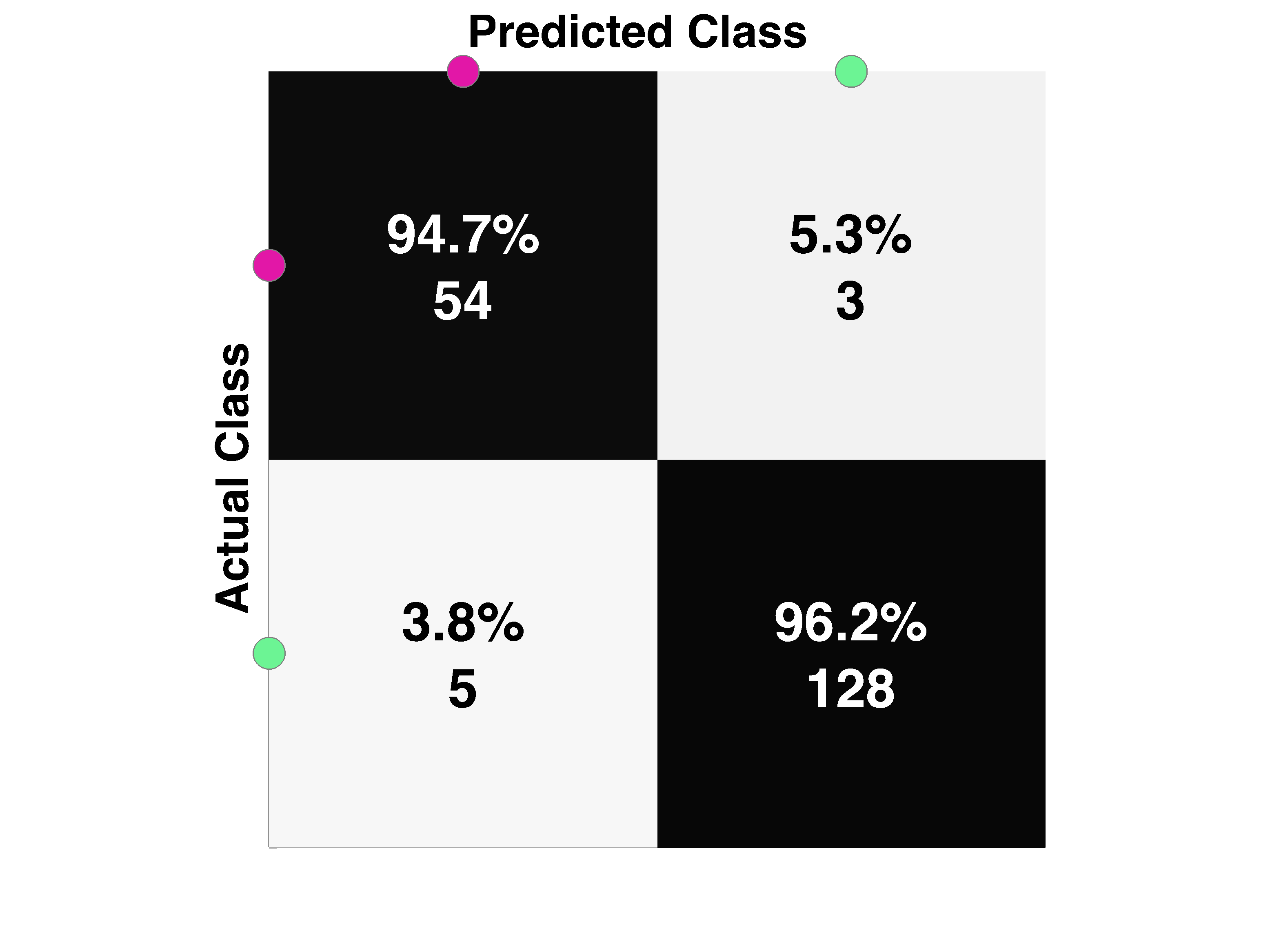


***C)***

Supplement: Supplementary file 3 — Multivariate statistical analysis of the cut model. a Unsupervised principal component analysis (PCA) analysis of the spectral differences (600–1000 m/z) between normal tissue compared to breast cancer using the cut electrosurgical modality. b Supervised linear discriminant analysis (LDA) plot comparing normal tissue to tumour using cut mode. c Confusion matrix demonstrating diagnostic accuracy of the cut model, following leave-one-patient-out cross-validation, with sensitivity (94.7%) and specificity (96.2%) (DOCX 367 kb). [file 13058_2017_845_MOESM3_ESM.docx]

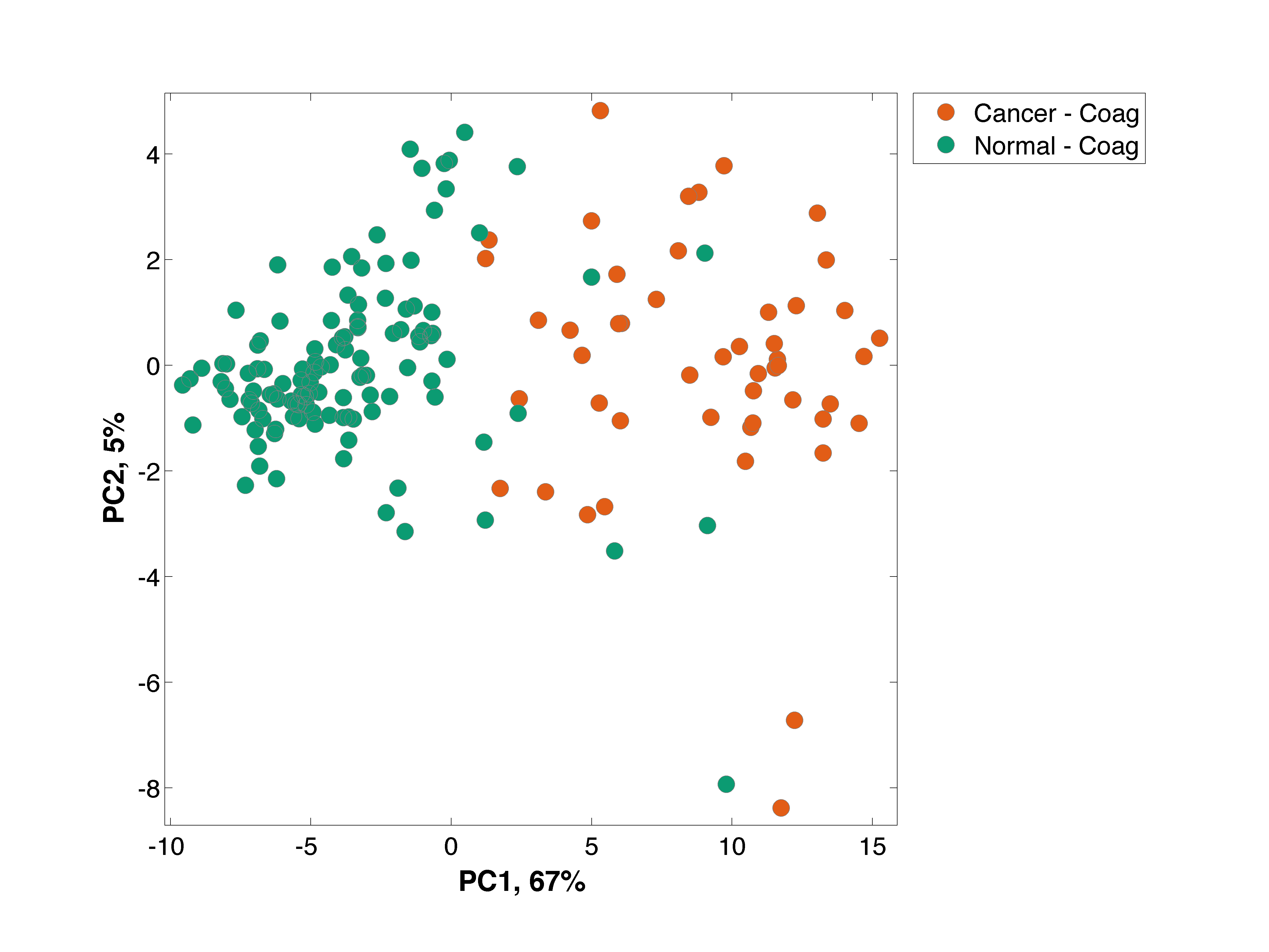


**a)**

***
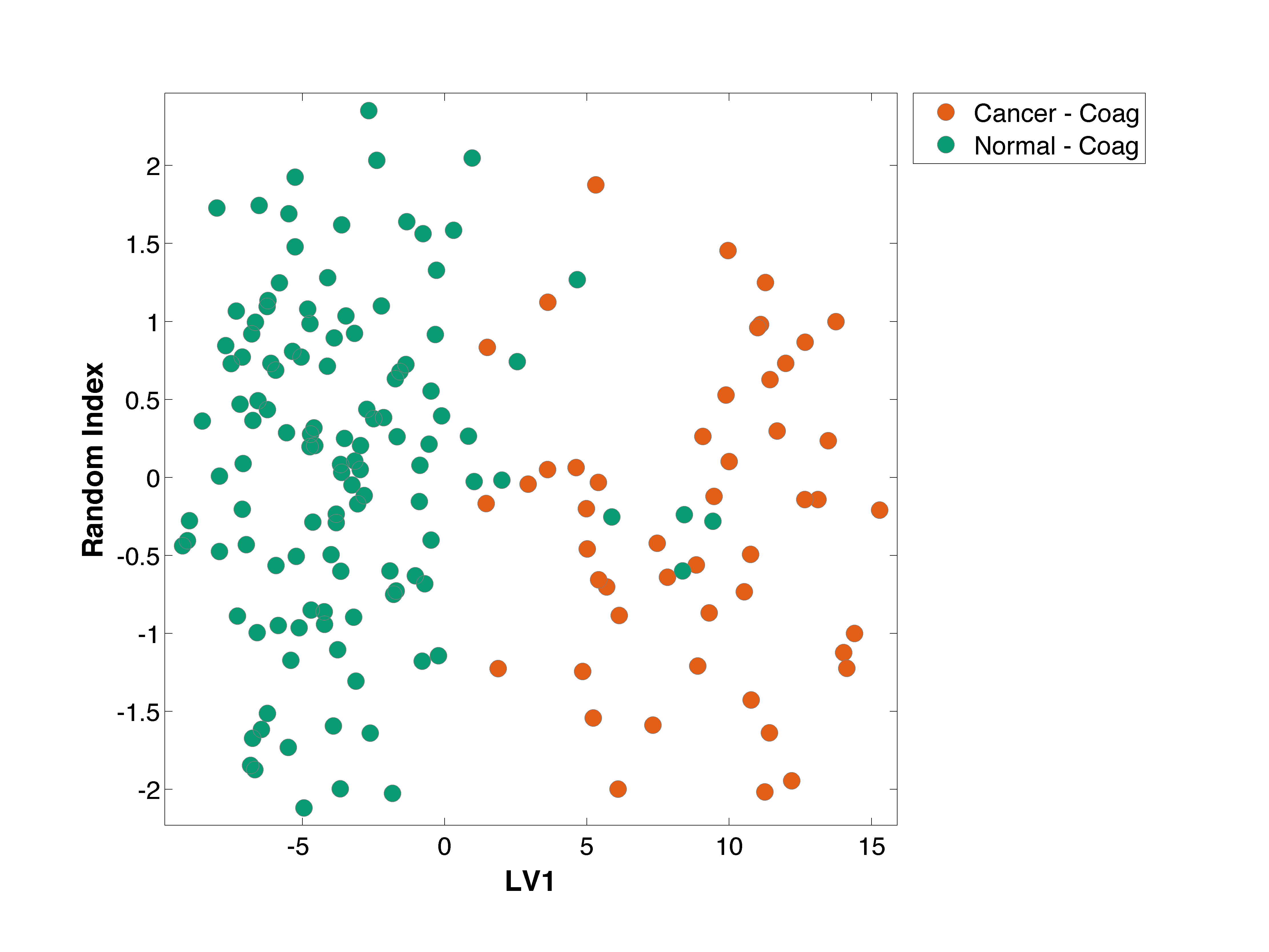
***

***b)***

***
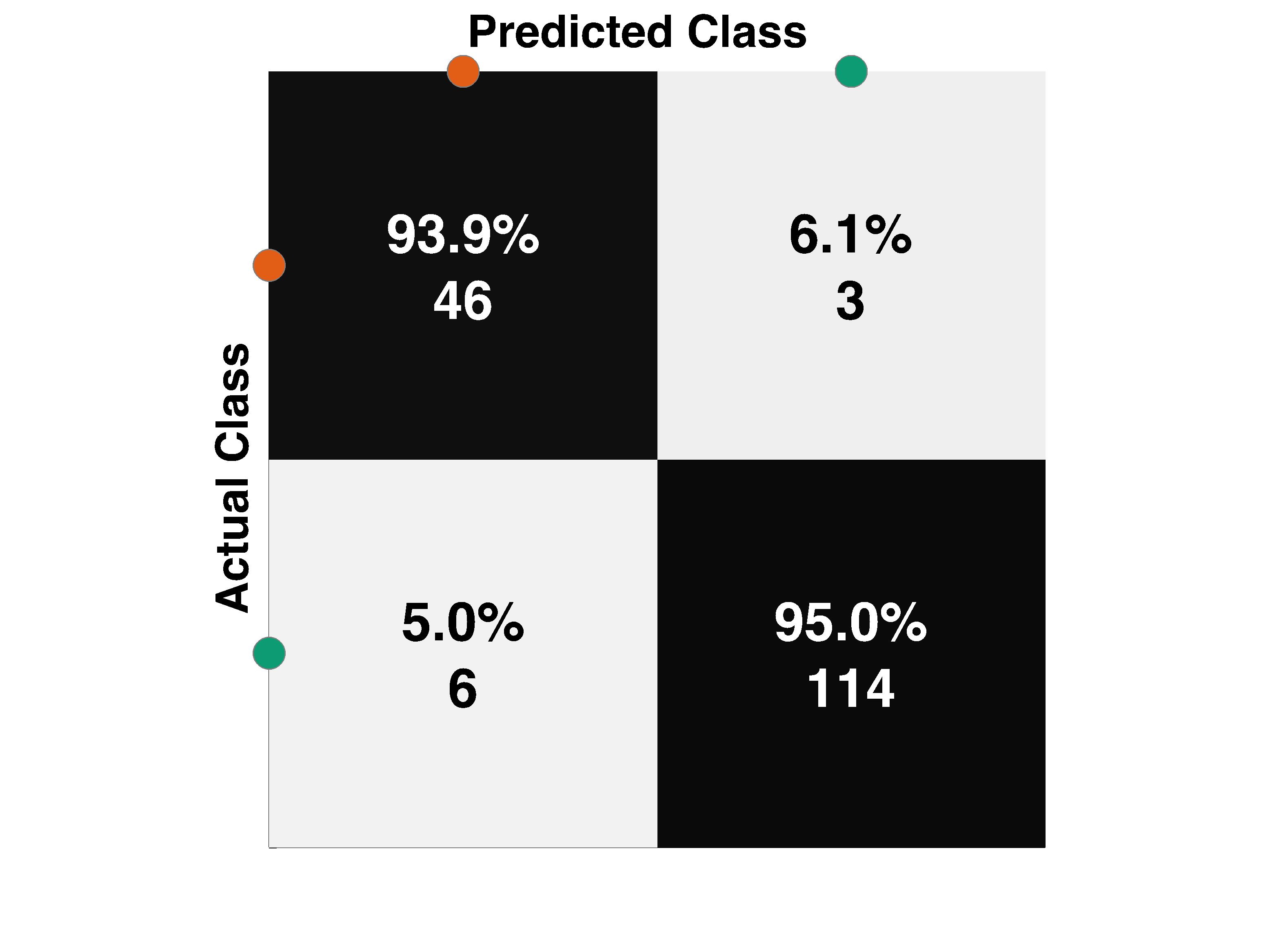
***

***c)***

Supplement: Supplementary file 4 — Multivariate statistical analysis of the coag model. a Unsupervised PCA analysis of the spectral differences (600–1000 m/z) between normal tissue compared to breast cancer using coag electrosurgical modality. b Supervised LDA plot comparing normal tissue to tumour using coag mode. c Confusion matrix demonstrating diagnostic accuracy of the coag model following leave-one-patient-out cross-validation, with sensitivity (93.9%) and specificity (95.0%) (DOCX 332 kb). [file 13058_2017_845_MOESM4_ESM.docx]

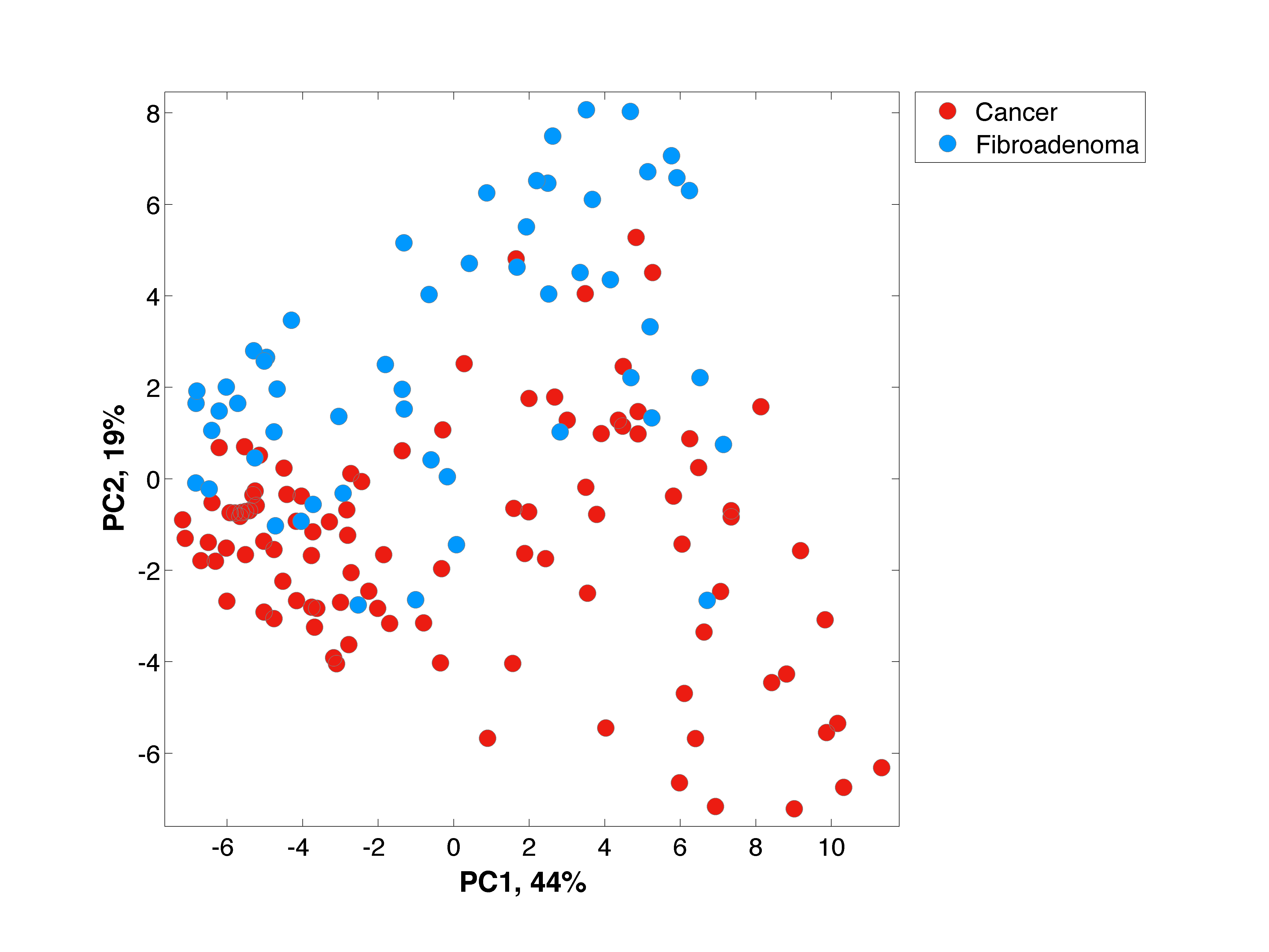


**a)**


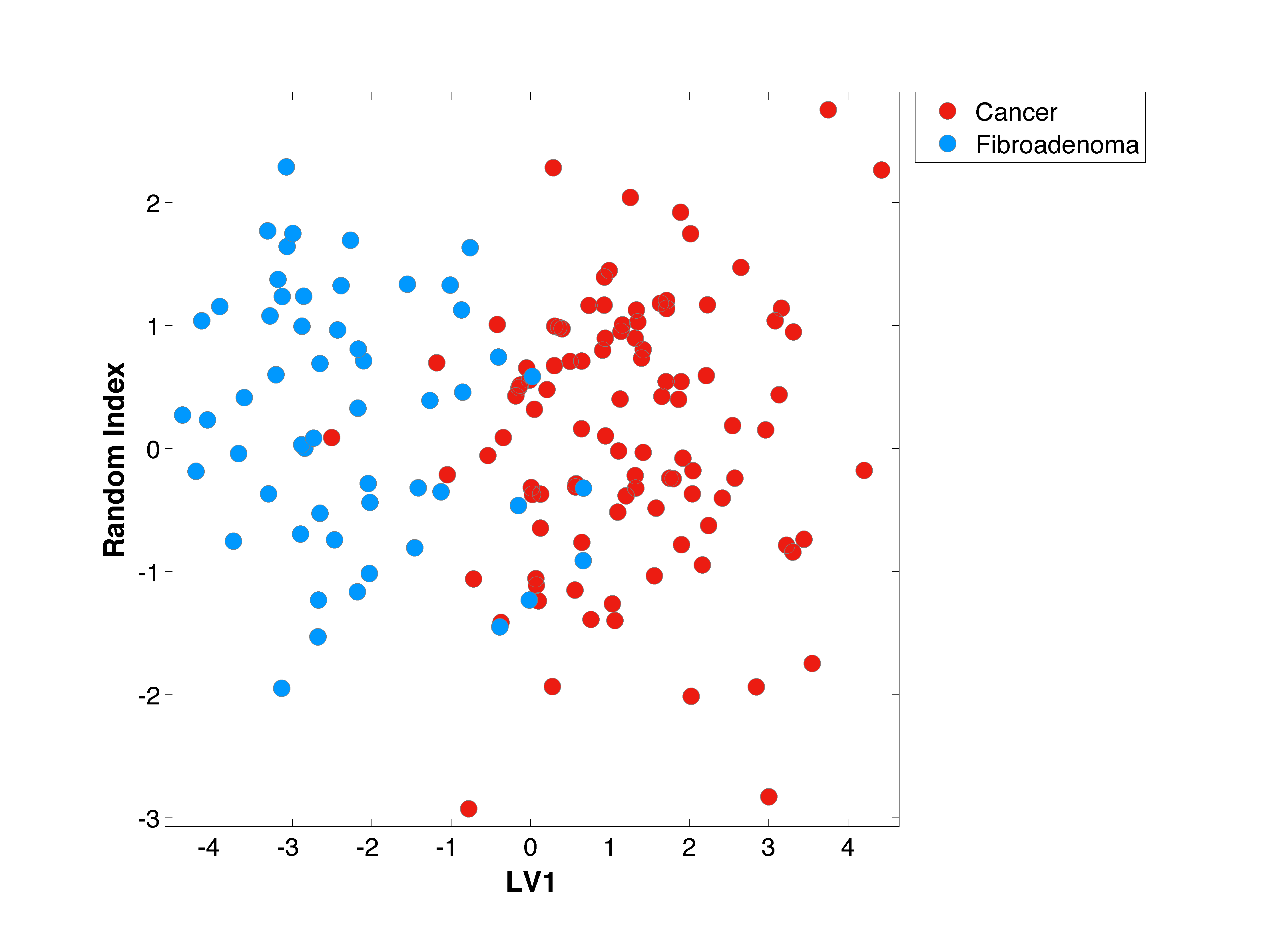


**b)**

**c)**
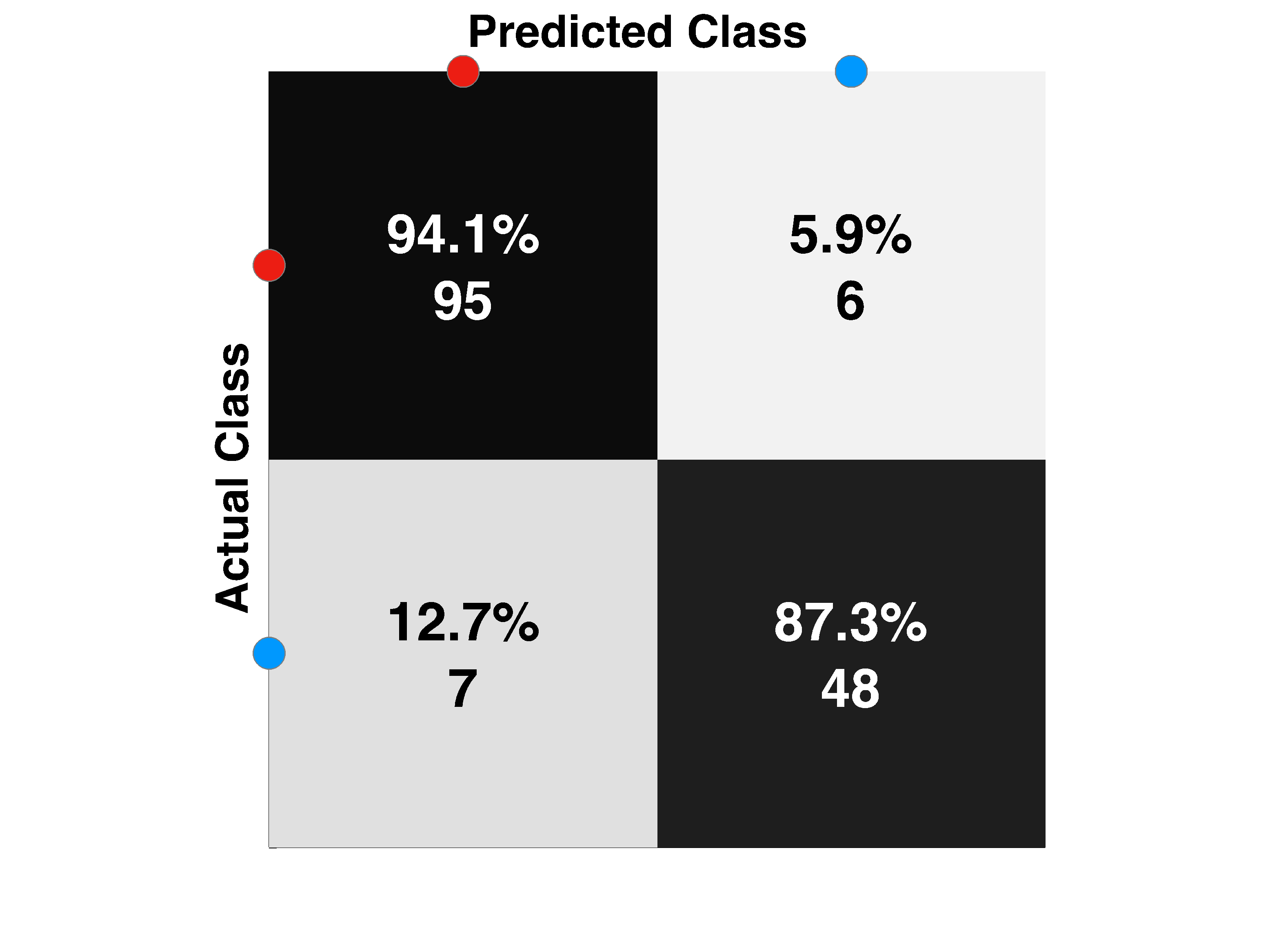

Supplement: Supplementary file 6 — Multivariate statistical analysis of fibroadenoma (B2) compared to cancer (B5b). a Unsupervised PCA analysis of the spectral differences (600–1000 m/z) between fibroadenoma samples compared to breast cancer using combined cut and coag electrosurgical modalities. b Supervised LDA plot comparing fibroadenoma to cancer using cut and coag modes. c Confusion matrix demonstrating diagnostic accuracy of the model: 55 solid fibroadenoma samples are compared to 101 tumour (B5b) samples. Sensitivity of tumour classification is high at 94.1% but specificity for the diagnosis of benign fibroadenoma is lower at 87.3% (DOCX 364 kb). [file 13058_2017_845_MOESM6_ESM.docx]
