# Supplementary material for: Rapid evaporative ionisation mass spectrometry of electrosurgical vapours for the identification of breast pathology: towards an intelligent knife for breast cancer surgery
Source: Breast Cancer Res. 2017 May 23;19:59. doi: 10.1186/s13058-017-0845-2 (PMC5442854; doi:10.1186/s13058-017-0845-2)
Supplement: Supplementary file 5 — Significant m/z peak differences in mean intensity between normal tissue and cancer, with fold changes and q value (false discovery rate (FDR)-corrected p value). Negative fold changes denote peaks that are lower in tumour compared with normal tissue (DOCX 48 kb). [file 13058_2017_845_MOESM5_ESM.docx]

| **m/z** | **Log_2_fold change** | **q-value** | **m/z** | | **Log_2_ fold change** | **q-value** |
| --- | --- | --- | --- | --- | --- | --- |
| 671.47 | 0.53 | 0.007 | 766.54 | 0.51 | | <0.001 |
| 673.48 | 0.59 | 0.002 | 768.55 | 0.62 | | <0.001 |
| 687.50 | 0.45 | <0.001 | 770.57 | 0.70 | | 0.031 |
| 699.50 | 0.63 | 0.007 | 772.58 | 0.45 | | <0.001 |
| 713.51 | 0.61 | 0.028 | 792.55 | 0.70 | | 0.023 |
| 714.51 | 0.31 | <0.001 | 794.57 | 0.53 | | <0.001 |
| 716.52 | 0.31 | <0.001 | 865.70 | -4.96 | | <0.001 |
| 717.51 | 0.54 | 0.009 | 891.72 | -6.02 | | <0.001 |
| 735.47 | 0.47 | 0.002 | 893.73 | -6.19 | | <0.001 |
| 742.54 | 0.63 | 0.015 | 917.73 | -4.72 | | <0.001 |
| 744.55 | 0.42 | <0.001 | 919.75 | -5.21 | | <0.001 |
| 747.51 | 0.54 | 0.001 | 921.76 | -5.35 | | <0.001 |
